# The Potential Benefits of Handling Mixture Statistics via a Bi‐Gaussian EnKF: Tests With All‐Sky Satellite Infrared Radiances

**DOI:** 10.1029/2022MS003357

**Published:** 2023-02-06

**Authors:** Man‐Yau Chan, Xingchao Chen, Jeffrey L. Anderson

**Affiliations:** ^1^ Department of Meteorology and Atmospheric Science The Pennsylvania State University University Park PA USA; ^2^ Center for Advanced Data Assimilation and Predictability Techniques The Pennsylvania State University University Park PA USA; ^3^ Data Assimilation Research Section Computational Information Systems Laboratory National Center for Atmospheric Research Boulder CO USA

**Keywords:** data assimilation, Gaussian mixture models, satellite observations, numerical weather prediction/forecasting, convection, ensembles

## Abstract

The meteorological characteristics of cloudy atmospheric columns can be very different from their clear counterparts. Thus, when a forecast ensemble is uncertain about the presence/absence of clouds at a specific atmospheric column (i.e., some members are clear while others are cloudy), that column's ensemble statistics will contain a mixture of clear and cloudy statistics. Such mixtures are inconsistent with the ensemble data assimilation algorithms currently used in numerical weather prediction. Hence, ensemble data assimilation algorithms that can handle such mixtures can potentially outperform currently used algorithms. In this study, we demonstrate the potential benefits of addressing such mixtures through a bi‐Gaussian extension of the ensemble Kalman filter (BGEnKF). The BGEnKF is compared against the commonly used ensemble Kalman filter (EnKF) using perfect model observing system simulated experiments (OSSEs) with a realistic weather model (the Weather Research and Forecast model). Synthetic all‐sky infrared radiance observations are assimilated in this study. In these OSSEs, the BGEnKF outperforms the EnKF in terms of the horizontal wind components, temperature, specific humidity, and simulated upper tropospheric water vapor channel infrared brightness temperatures. This study is one of the first to demonstrate the potential of a Gaussian mixture model EnKF with a realistic weather model. Our results thus motivate future research toward improving numerical Earth system predictions though explicitly handling mixture statistics.

## Key Points

- Current ensemble data assimilation (DA) methods assume that forecasts follow a normal distribution. This assumption is often invalid
- In this study, we propose a computationally efficient ensemble DA method that handles clear and cloudy forecasts separately
- This study uses a realistic weather model (WRF) to show that this method can outperform the EnKF

## 1 Introduction

Earth system analysis and forecasting systems rely on ensemble data assimilation (ensemble DA, or EDA) methods to convert observations into corrections for Earth system model variables (ECMWF, 2016; Edwards et al., 2015; Helmert et al., 2018; Hersbach et al., 2020; Keppenne et al., 2005; Park & Xu, 2016; Reichle et al., 2009; Stammer et al., 2016). Current operational EDA methods typically assume that every member in an input forecast ensemble is drawn from a distribution only containing a single Gaussian kernel (i.e., a Gaussian distribution; henceforth termed the unmixed ensemble assumption; e.g., Geer et al. (2018) and Dowell et al. (2022)). The effectiveness of such methods can thus be limited by the validity of this assumption.

The unmixed ensemble assumption is violated for ensembles that are uncertain about the presence or absence of clouds at any model grid point. This is because clear atmospheric columns and cloudy atmospheric columns often have different dynamic, thermodynamic, and radiative properties (e.g., Emanuel (1994) and Markowski and Richardson (2010)). Cloudy statistics are thus often different from clear statistics (e.g., Grimes and Pardo‐Igúzquiza (2010) and Geer and Bauer (2011)). If some ensemble members are cloudy at a location, and other members are clear at this location, the ensemble can exhibit mixed statistics (Chan, Anderson, & Chen, 2020; Harnisch et al., 2016; Honda et al., 2018; Minamide & Zhang, 2017). More evidence of mixed statistics can be found in Supporting Information S1. Though current EDA methods have been remarkably successful at assimilating cloud‐affected satellite radiance observations (e.g., F. Zhang et al. (2016), Geer et al. (2018), Chan, Zhang, et al. (2020), Jones et al. (2020), Li et al. (2021), Mallick and Jones (2022), M. Zhang et al. (2022), Chan and Chen (2022), Hartman et al. (2021), and Chan, Chen, and Leung (2022), Chandramouli et al. (2022)) the effectiveness of current operational EDA methods is likely limited when the ensemble statistics are mixed.

This limitation can be mitigated by extending current operational EDA methods to handle mixed statistics. One possibility is to extend the commonly used ensemble Kalman filter, or the EnKF (Anderson, 2003; Burgers et al., 1998; Edwards et al., 2015; Evensen, 1994; Helmert et al., 2018; P. L. Houtekamer & Mitchell, 1998; Hunt et al., 2007; Keppenne et al., 2005; Park & Xu, 2016; Reichle et al., 2009; Stammer et al., 2016; Tippett et al., 2003; Whitaker & Hamill, 2002), to handle members drawn from forecast distributions with two Gaussian kernels. Specifically, we assume that forecast members that are clear at an observation site (henceforth, clear members) are drawn from one Gaussian kernel, and forecast members that are cloudy at this site (henceforth, cloudy members) are drawn from a different Gaussian kernel. This bi‐Gaussian extension of the EnKF (henceforth, the BGEnKF) allows the clear ensemble statistics to be handled separately from the cloudy ensemble statistics (Chan, Anderson, & Chen, 2020), thus addressing the issue of mixed statistics.

We recently proposed a computationally efficient BGEnKF to handle mixtures of clear and cloudy members (Chan, Anderson, and Chen (2020); henceforth, the CAC20 BGEnKF). Unlike similar methods proposed in the past (Dovera & Della Rossa, 2011; Reich, 2012; Sondergaard & Lermusiaux, 2013a, 2013b), the CAC20 BGEnKF does not use an expectation maximization (EM) algorithm to estimate the mean and covariances of the two Gaussian kernels. Instead, the CAC20 BGEnKF assigns the sample mean and covariances of the cloudy members to one Gaussian kernel, and those of the clear members to the other Gaussian kernel. This assignment circumvents the computational cost and issues associated with using the EM algorithm in high dimensional spaces (see Chan, Anderson, and Chen (2020) for more information). Furthermore, the CAC20 BGEnKF converts clear members into cloudy members, or vice versa, without involving the costly square‐root computations or Cholesky decompositions of high‐dimensional forecast covariance matrices.

The purpose of this study is to demonstrate that a variant of the CAC20 BGEnKF can outperform the EnKF using a realistic high‐order weather model (the Weather Research and Forecast model; WRF). To do so, this new BGEnKF is implemented into the state‐of‐the‐art Pennsylvania State University EnKF system (henceforth, the PSU‐EnKF system; Meng and Zhang (2007), Meng and Zhang (2008), and Chan, Zhang, et al. (2020)). This demonstration is done using perfect model observing system simulation experiments (OSSEs) of a case of tropical convection over the equatorial Indian Ocean. This case occurred during the onset of the active phase of the October 2011 Madden‐Julian Oscillation event (MJO; Madden and Julian (1971), Madden and Julian (1972), and S. Wang et al. (2015)).

The structure of this paper is as follows. In Section 2, we will give an overview of the BGEnKF algorithm, discuss how clear and cloudy members are identified, and modifications made to the CAC20 BGEnKF algorithm. A detailed description of the current BGEnKF, along with suggestions on handling more than two Gaussian kernels, can be found in Supporting Information S1. Following that, we will discuss the setup of our OSSEs in Section 3 and the results in Section 4. We will then conclude in Section 5.

## 2 On the BGEnKF Algorithm

### 2.1 On the Identification of Clear and Cloudy Members

The BGEnKF requires identifying clear and cloudy members at each iteration of the serial data assimilation loop. A simple identification method is to check if the members' column‐integrated liquid and/or frozen water mass contents exceed a threshold.

The choice of which phase of water to include in the column integration depends on the specifics of the forecast model. As will be discussed in Section 3.3, this study used a WRF model setup with a 9‐km horizontal grid spacing and without convective parameterization. This WRF model setup cannot realistically resolve trade cumuli since the typical width of trade cumuli is ∼1‐km. As such, we consider columns with trade cumuli and entirely cloud‐free columns as clear member columns, and the remaining members as cloudy member columns. Since trade cumuli do not typically grow above the melting layer (Johnson et al., 1999), clear members do not possess frozen water. It thus seems appropriate to use column‐integrated ice mass content (*ξ*) to distinguish between clear and cloudy member columns. To be precise, we compute *ξ* at a given model column via

(1) $$\xi \equiv \int_{0}^{z_{\mathrm{top}}}\rho \left(q_{i}+q_{s}+q_{g}\right)dz$$

where *z*_*top*_ is the model top altitude and *ρ* represents air density. Furthermore, *q*_*i*_, *q*_*s*_, and *q*_*g*_ are the mass mixing ratios of ice, snow and graupel, respectively.

In this study, we will consider model columns with *ξ* ≥ 1 g/m^2^ as cloudy, and model columns with *ξ* < 1 g/m^2^ as clear. The cloudy and clear infrared window channel simulated brightness temperature statistics (Window‐BT; central wavelength of 10.5 μm) do not vary noticeably for model column *ξ* thresholds between 0.8 and 1.2 g/m^2^. Future studies can refine the threshold value or seek better ways to separate clear and cloudy column members.

### 2.2 Overview of the BGEnKF Algorithm

This study's BGEnKF (and the CAC20 BGEnKF) assimilates observations with Gaussian observation likelihoods under the assumption that clear members are drawn from one Gaussian kernel and cloudy members are drawn from another Gaussian kernel. Suppose we seek to constrain the following extended state vector ψ

(2) $$\psi \equiv \left[\begin{array}{c} x\\ h\left(x\right)\\ \xi \left(x\right) \end{array}\right]$$

where x represents the model state, $h\left(x\right)$ represents applying the observation operator h on x, and $\xi \left(x\right)$ represents computing *ξ* at all observation sites (Equation 1). Note that observation sites here refers to the latitude and longitude of the observation (i.e., the vertical position is not considered for now). Supposing there are *N*_*x*_ elements in x and *N*_*y*_ observations, then ψ has *N*_*x*_ + 2*N*_*y*_ elements.

The BGEnKF assumes that the prior probability density function [pdf; $p\left(\psi \right)$] can be represented by the bi‐Gaussian pdf

(3) $$p\left(\psi \right)=w_{\text{clr}}^{f}\;G\left(\psi ;\;\;\bar{\psi_{\text{clr}}^{f}},P_{\text{clr}}^{f}\right)+w_{\text{cld}}^{f}\;G\left(\psi ;\;\;\bar{\psi_{\text{cld}}^{f}},P_{\text{cld}}^{f}\right).$$

The subscript “clr” denotes clear cluster quantities, and the subscript “cld” denotes cloudy cluster quantities. $G\left(\psi ;\;\;\bar{\psi_{\text{clr}}^{f}},P_{\text{clr}}^{f}\right)$ denotes the clear cluster's Gaussian kernel with mean state $\bar{\psi_{\text{clr}}^{f}}$ and covariance matrix $P_{\text{clr}}^{f}$. Similarly, $G\left(\psi ;\;\;\bar{\psi_{\text{cld}}^{f}},P_{\text{cld}}^{f}\right)$ denotes the cloudy cluster's Gaussian kernel with mean state $\bar{\psi_{\text{cld}}^{f}}$ and covariance matrix $P_{\text{cld}}^{f}$. The scalar quantities $w_{\text{clr}}^{f}$ and $w_{\text{cld}}^{f}$ are the respective weights of the clear and cloudy Gaussian kernels. Note that

$$\begin{array}{ccccc} w_{\text{clr}}^{f}+w_{\text{cld}}^{f}=1, & & w_{\text{clr}}^{f}\geq 0, & \;\text{and,}\; & w_{\text{cld}}^{f}\geq 0. \end{array}$$

The various parameters in Equation 3 can be estimated by the procedure described in CAC20 or in the supporting information.

Upon assimilating an observation $y^{o}$ with Gaussian observation error, the BGEnKF produces an ensemble that is consistent with the analysis pdf

(4) $$p\left(\psi |y^{o}\right)=w_{\text{clr}}^{a}\;G\left(\psi ;\;\;\bar{\psi_{\text{clr}}^{a}},P_{\text{clr}}^{a}\right)+w_{\text{cld}}^{a}\;G\left(\psi ;\;\;\bar{\psi_{\text{cld}}^{a}},P_{\text{cld}}^{a}\right).$$

Here, $w_{\text{clr}}^{a}$ and $w_{\text{cld}}^{a}$ are the respective analysis weights of clear and cloudy Gaussian kernels, $\bar{\psi_{\text{clr}}^{a}}$ and $\bar{\psi_{\text{cld}}^{a}}$ are the respective analysis means of the clear and cloudy Gaussian kernels, and $P_{\text{clr}}^{a}$ and $P_{\text{cld}}^{a}$ are the respective analysis covariances of the clear and cloudy Gaussian kernels. See CAC20 [or the supporting information] for the equations relating the analysis pdf's parameters to the forecast pdf's parameters.

Note that $w_{\text{clr}}^{f}$ is the probability that a prior member is clear, and $w_{\text{cld}}^{f}$ is the probability that a prior member is cloudy. Similarly, $w_{\text{clr}}^{a}$ is the probability that a posterior member is clear, and $w_{\text{cld}}^{a}$ is the probability that a posterior member is cloudy. We estimate $w_{\text{clr}}^{f}$ by counting the number of clear members, and likewise for $w_{\text{cld}}^{f}$. To generate a posterior ensemble that is consistent with $w_{\text{clr}}^{a}$ and $w_{\text{cld}}^{a}$, the BGEnKF will shift members between the clear and cloudy clusters (see later).

**Figure 1:**
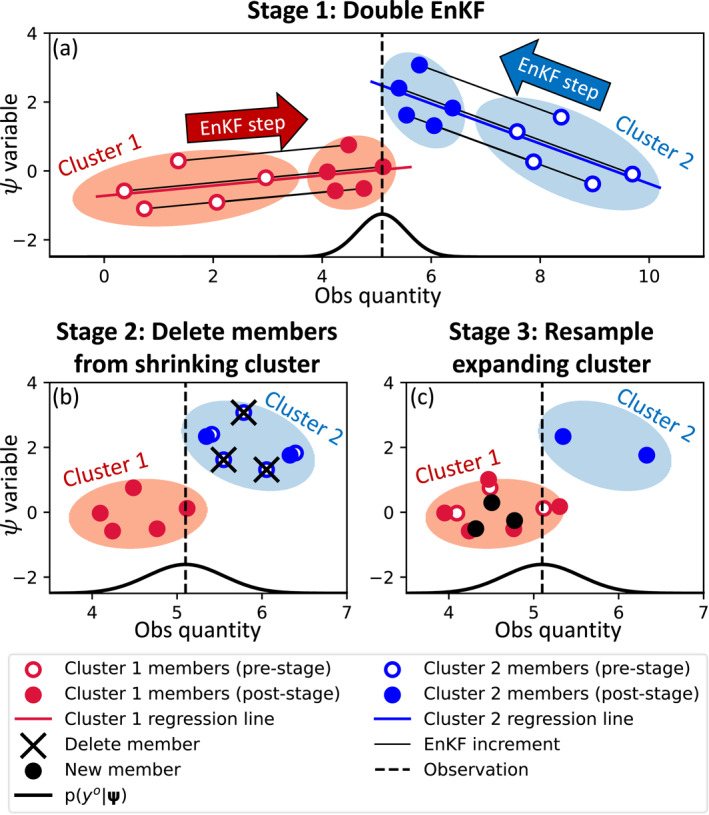
A bivariate demonstration of the three‐stage process of the bi‐Gaussian extension of the ensemble Kalman filter (BGEnKF) algorithm. The light red ovals highlight cluster 1 members and the light blue ovals highlight cluster 2 members. Prior to running the BGEnKF update, the prior members have already been separated into two clusters. The BGEnKF's first stage is to employ the ensemble Kalman filter update equations on the two clusters separately (panel a). In the second stage (panel b), the BGEnKF identifies the shrinking cluster (the blue cluster 2 in this case), deletes an appropriate number of members from this cluster, and adjusts the remaining members to prevent the deletion from changing this cluster's mean. The BGEnKF's final stage (panel c) is to recreate the deleted members by resampling from the expanding cluster (cluster 1).

The BGEnKF converts a forecast ensemble into an analysis ensemble through a three‐stage process (illustrated in Figure 1). First, two EnKF procedures are executed (Figure 1a): once for clear members using clear forecast statistics $\left(\bar{\psi_{\text{clr}}^{f}},P_{\text{clr}}^{f}\right)$, and a second time for cloudy members using cloudy forecast statistics $\left(\bar{\psi_{\text{cld}}^{f}},P_{\text{cld}}^{f}\right)$. Afterward, to reflect the update to the bi‐Gaussian pdf weights, clear members will be replaced with cloudy members, or vice versa. For example, if the BGEnKF increased the weight on the clear Gaussian distribution (i.e., $w_{\text{clr}}^{a}>w_{\text{clr}}^{f}$ and $w_{\text{cld}}^{a}<w_{\text{cld}}^{f}$), some cloudy members will be replaced with clear members. This is achieved by deleting some cloudy members (Figure 1b) and replacing the deleted members with resampled clear members (Figure 1c). Once these three stages are completed, the ensemble obeys Equation 4. See the supporting information for a detailed description of these three stages.

The deleting (Figure 1b) and resampling (Figure 1c) processes are as follows. Reusing the previous paragraph's example, we first compute the number of cloudy members to be deleted, $N_{\text{del}}\equiv \text{round}\left(\left(w_{\text{cld}}^{f}-w_{\text{cld}}^{a}\right)N_{E}\right)$. The $\text{round}\left(\cdot \right)$ operation here rounds ⋅ to the nearest integer. The *N*_del_ cloudy members with the smallest ensemble‐simulated observation perturbations are then deleted. We then construct *N*_del_ new clear members through linear combinations of the existing clear members (i.e., we resample the clear members). These weights are encoded in a resampling matrix **T**. See Text S4 of the Supporting Information S1 for the details of **T**.

Similar deleting and resampling procedures are used in situations where clear members are deleted and cloudy members are resampled. We first compute the number of clear members to be deleted, $N_{\text{del}}\equiv \text{round}\left(\left(w_{\text{clr}}^{f}-w_{\text{clr}}^{a}\right)N_{E}\right)$. The *N*_del_ clear members with the smallest ensemble‐simulated observation perturbations are then deleted. Afterward, *N*_del_ new cloudy members are created through linear combinations of the existing clear members using **T**.

### 2.3 Revised Extended State Formulation for Better Scalable Parallelism

The most important modification to the original CAC20 BGEnKF lies in the definition of ψ. The CAC20 BGEnKF's ψ only contains x and a single observation. As such, the CAC20 BGEnKF algorithm is a sequential algorithm that scales inefficiently with parallelization on high latency clusters (Anderson & Collins, 2007). For more efficient scaling with parallelization, this study's ψ (i.e., Equation 2) contains all of the information necessary to assimilate all observations (Anderson & Collins, 2007).

Since the definition of ψ has been modified, we will redefine our forecast ensemble. Supposing an ensemble size of *N*_*E*_, the forecast ψ ensemble is constructed by evaluating

(5) $$\psi_{n}^{f}\equiv \left[\begin{array}{c} x_{n}^{f}\\ h\left(x_{n}^{f}\right)\\ \xi \left(x_{n}^{f}\right) \end{array}\right]\;\;\;\;\forall \;\;n=1,2,\text{…},N_{E}$$

where $\psi_{n}^{f}$ is the ψ of the *n* − th forecast member, and $x_{n}^{f}$ is the x of the same forecast member.

The revised formulation enhances the scalability of the BGEnKF by avoiding evaluations of $h\left(x\right)$ and $\xi \left(x\right)$ at each iteration of the serial assimilation loop. This is because such evaluations may require costly inter‐process communications. The removal of such evaluations is achieved through two modifications to the CAC20 BGEnKF. First, the assimilation of an observation uses the BGEnKF update equations (see CAC20 or the Supporting Information S1) to update all model state elements, all simulated observation state elements and all *ξ* elements in the forecast ensemble. The CAC20 BGEnKF, in contrast, updates all model state elements and only a single simulated observation state element. This difference in updates leads to a second modification: to assimilate the *m* − th observation, instead of evaluating $h\left(x\right)$ and $\xi \left(x\right)$, this study's BGEnKF only needs to read the corresponding simulated observation and the *ξ* values from ψ.

### 2.4 Revised Expanding Cluster Resampling Procedure

The other major change to the CAC20 BGEnKF lies in the resampling matrix ***T***. ***T*** is used to resample the Gaussian kernel that better agrees with the assimilated observation, thus representing the increase in the weight of this kernel. The CAC20 BGEnKF uses a stochastic procedure to construct ***T*** (see Equation 18 and Appendix B of CAC20). Unfortunately, because random number generators are involved, the analysis ensemble generated on one computing cluster cannot be easily replicated on another computing cluster.

To ensure the replicability of the BGEnKF's analysis ensembles, we replaced the stochastic component of the CAC20 BGEnKF's ***T*** (***W*** in Appendix B of Chan, Anderson, and Chen (2020)) with a deterministic one. Supposing that we want to add *N*_new_ cloudy members to the ensemble to represent an increased weight of the cloudy Gaussian distribution, the new deterministic ***W*** is defined as

(6) $$W\equiv \left[\begin{array}{cc} I_{N_{new}^{*}} & 0_{N_{new}^{*}\times \left(N_{new}-N_{new}^{*}\right)} \end{array}\right]-\frac{1}{N_{\text{new}}}1_{N_{\mathrm{new}}^{*}\times N_{\mathrm{new}}}$$

where

(7) $$N_{\text{new}}^{*}\equiv \left\{\begin{array}{c} \begin{array}{cc} N_{\text{new}}-1 & \forall \;N_{\text{new}}\leq N_{\text{pre}}\\ N_{\text{pre}} & \text{otherwise} \end{array}\; \end{array}\right..$$

The integer $N_{\text{new}}^{*}$ is the number of pre‐resampling cloudy members that will be linearly combined to form *N*_new_ new cloudy members and *N*_pre_ is the number of cloudy members at the start of the resampling procedure. Furthermore, $I_{N_{new}^{*}}$ is an $N_{\text{new}}^{*}\times N_{\text{new}}^{*}$ identity matrix, $0_{N_{new}^{*}\;\times \;\left(N_{new}\;-\;N_{new}^{*}\right)}$ is an $N_{\text{new}}^{*}\times \left(N_{\text{new}}-N_{\text{new}}^{*}\right)$ matrix of zeros, and $1_{N_{\mathrm{new}}^{*}\;\times \;N_{\mathrm{new}}}$ is an $N_{\text{new}}^{*}\times N_{\text{new}}$ matrix of ones. Note that Equation 6 is also applied in the situation where *N*_new_ clear members are being added to the ensemble. A detailed description of the revised resampling procedure is provided in the supporting information.

An interesting property of Equation 6 is that the resulting **T** is a mostly diagonal matrix. Specifically, nearly all of the off‐diagonal elements in **T** are either zero or much smaller than the diagonal elements (not shown). As a result, the resampled members are essentially copies of the pre‐resampling members, plus some small perturbation. The CAC20 stochastic **W** formulation does not have this property. Future work can investigate how the BGEnKF's behavior changes with different **W** formulations.

Note that the resampling process is designed to preserve multivariate relationships in the expanding cluster. All linear multivariate relationships are completely preserved and nonlinear multivariate relationships are likely mostly preserved. The perfect preservation of linear multivariate relationships is a result of perfectly preserving the expanding cluster's covariance matrix during resampling. The multivariate nonlinear relationships are likely to be well preserved (albeit imperfectly) because new members are generated by copying existing expanding cluster members and adding small adjustments.

### 2.5 Heuristic Measures

#### 2.5.1 Localization

The BGEnKF is likely more susceptible to sampling noise than the EnKF because the sample size used to estimate each cluster's mean state and Kalman gain are smaller than the sample size used to estimate the mean state and covariance matrix of the entire ensemble. As such, we employ two heuristic measures that are similar to those of CAC20. First, we spatially localize the BGEnKF analysis increment using the Gaspari‐Cohn fifth order polynomial (GC99; Gaspari and Cohn (1999)). If ***ρ*** represents a vector of GC99 localization factors, we construct the localized updated extended state vector for member *n* via

(8) $$\psi_{n}^{a}\leftarrow \rho ◦\left(\psi_{n}^{a}-\psi_{n}^{f}\right)+\psi_{n}^{f}$$

where ◦ represents element‐wise multiplication. In the cases where either $w_{\text{clr}}^{f}=1$ or $w_{\text{cld}}^{f}=1$ (i.e., the bi‐Gaussian prior p.d.f. turns Gaussian), this localization method is identical to Kalman gain localization (e.g., Anderson et al. (2009), Meng and Zhang (2008), Whitaker et al. (2008), and P. L. Houtekamer and Zhang (2016)).

Note that this localization method (Equation 8) localizes the impacts of replacing clear members with cloudy members (or vice versa). As an example, suppose the BGEnKF replaces a cloudy forecast member with a clear analysis member. The localization process (Equation 8) first computes the difference between the cloudy forecast member and the clear analysis member (i.e., the member's change due to the BGEnKF). This difference is then localized and applied to the cloudy forecast member. The resulting member follows the clear analysis member at the observation site and becomes increasingly like the cloudy forecast member with increasing distance from the observation site. Future work can examine other approaches to localize the impacts of deleting and replacing ensemble members.

#### 2.5.2 Handling Overly Small Clusters

The second heuristic sampling error mitigation measure is to switch from using the BGEnKF to using the EnKF whenever the pre‐resampling expanding cluster is too small (*N*_pre_ < 0.8*N*_*E*_, where *N*_*E*_ is the ensemble size), or whenever any cluster is too small (less than 0.1*N*_*E*_). A similar heuristic measure is used in CAC20.

#### 2.5.3 Mitigating Unphysical Weight Updates

Another issue specific to the BGEnKF is its occasional tendency to generate unphysical weight updates. Specifically, the BGEnKF occasionally expands the clear cluster when a cloudy observation is assimilated, and vice versa. This is because the BGEnKF does not explicitly consider whether an observation is clear or cloudy when assimilating it.

As an example, suppose the clear‐sky cluster's Window‐BT values have a mean of 285 K and variance of 3^2^ K^2^, and the cloudy cluster's Window‐BT values have a mean of 270 K and a variance of 20^2^ K^2^. Furthermore, suppose half of the ensemble members are clear (i.e., $w_{\text{clr}}^{f}=0.5$), half of the ensemble members are cloudy (i.e., $w_{\text{cld}}^{f}=0.5$), a clear‐sky Window‐BT value of 295 K is observed, and the observation error variance is 3^2^ K^2^. Evaluating Bayes' rule (Text S3 in Supporting Information S1) yields $w_{\text{clr}}^{a}\approx 0.36$ and $w_{\text{cld}}^{a}\approx 0.64$. In other words, though a clear observation is assimilated, the clear cluster has shrunk and the cloudy cluster has expanded.

The BGEnKF is automatically switched to the EnKF whenever an unphysical weight update is detected. To do so, we first identify the whether the observation to be assimilated is definitively clear or cloudy. In the case of Window‐BT values over tropical ocean, observation values warmer than 290 K are definitively clear, and observation values cooler than 280 K are definitively cloudy. If the observation is definitively clear, but the cloudy cluster is expanded by the BGEnKF, or vice versa, the BGEnKF will switch over to the EnKF.

## 3 Materials and Methods

### 3.1 Description of October 2011 Tropical Convection Case

The BGEnKF was tested against the EnKF using a case of tropical convection over the equatorial Indian Ocean during the October 2011 MJO. This case is chosen because it can be reasonably replicated by regional WRF models (Chan, Zhang, et al., 2020; X. Chen, Pauluis, & Zhang, 2018; X. Chen & Zhang, 2019; Fu et al., 2017; S. Wang et al., 2015; Ying & Zhang, 2017, 2018; F. Zhang et al., 2017).

Our experiments are conducted over a 3 day period during the onset of this MJO event (15 October 2011–18 October 2011). Two persistent regions of enhanced convection (henceforth, “convective regions”) are observed in the 4‐km Global IR Data set of Janowiak et al. (2017) [henceforth, the MERG data set]. The first convective region (blue rectangle) occurs between 60°E and 75°E and persists beyond the 3‐day period. Westward propagation is observed in some of the clouds in this region, most notably between 1200 UTC on 16 October and 0000 UTC on 18 October. The second convective region (blue oval) appears on the eastern edge of the study domain at 1200 UTC on 16th October and exhibits a westward propagation that is similar to that of the first system. We will later assess our OSSE's nature run simulation by checking the nature run against these two convective regions.

### 3.2 Setup of WRF Model

**Figure 2:**
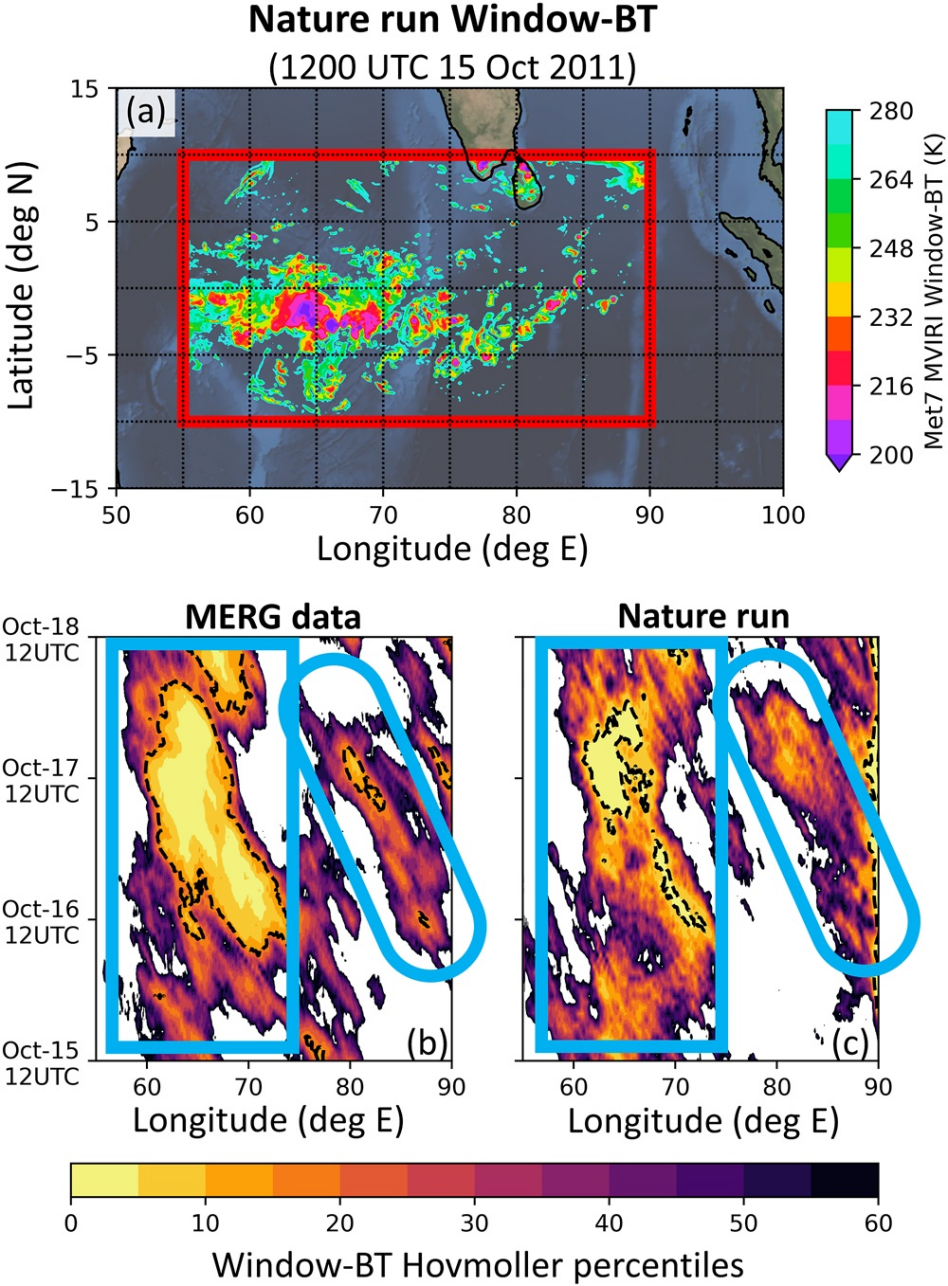
(a) Plot of our observing system simulated experiment domain overlaid with the nature run's simulated Window‐BT field at 1200 UTC on 15th October 2011. The red box in panel (a) indicates our study domain. Also shown are longitude‐time diagrams for the MERG data set (b) and nature run (c). In panels (b) and (c), the shadings indicate Window‐BT Hovmoller percentile values. These Window‐BT Hovmoller percentile values are constructed by first averaging Window‐BT values between 10°S and 10°N at every hour to produce a time‐longitude array of latitudinally averaged Window‐BT values. These arrays are then converted into percentiles before producing the longitude‐time percentile values. Note that the dashed black contours in (b) and (c) indicate areas where the time‐longitude arrays of latitudinally averaged Window‐BT values are below 260 K.

The Advanced Research version of the WRF model version 3.8 (Skamarock et al., 2008; University Corporation for Atmospheric Research, 2016) is used in this study. Following Chan, Zhang, et al. (2020), we construct a WRF domain over the study domain (red box in Figure 2a) with 432 × 243 horizontal grid points, 9‐km horizontal grid spacing, and 45 model levels. The bottommost 9 levels are within the lowest 1‐km of the atmosphere and the pressure level at the top of the domain is set to 20 hPa. The WRF integration time step is set to 20 s.

Our WRF model setup uses the following parameterization schemes. Cloud microphysical processes are handled by the WRF double‐moment 6‐class scheme (WDM6) proposed by Lim and Hong (2010). The updated Goddard shortwave scheme of Chou and Suarez (1999) and the Rapid Radiative Transfer Model (Global Circulation Model version; RRMTG) longwave scheme of Iacono et al. (2008) are used to parameterize radiative processes. The unified Noah land surface physics scheme (F. Chen & Dudhia, 2001) handles surface process and the Yonsei University (YSU) boundary layer scheme (Hong et al., 2006) is employed. No cumulus parameterization is employed because many studies have demonstrated that the 9‐km grid spacing is sufficient to resolve tropical mesoscale convective systems over the region (Chan & Chen, 2022; Chan, Zhang, et al., 2020; X. Chen, Leung, Feng, & Song, 2021; X. Chen, Leung, Feng, Song, & Yang, 2021; X. Chen, Pauluis, Leung, & Zhang, 2018; X. Chen, Pauluis, & Zhang, 2018; X. Chen & Zhang, 2019; X. Chen et al., 2020; X. Chen et al., 2022; S. Wang et al., 2015; Ying & Zhang, 2017, 2018; F. Zhang et al., 2017).

Note that the WDM6 microphysics scheme may be less optimal than other microphysics schemes at handling tropical cloud processes. Future work can investigate this possibility by assessing the performance of different microphysics schemes with regards to simulating tropical cloud processes (e.g., Jones et al. (2018)).

### 3.3 Setup of WRF Ensemble and Nature Run

This study's WRF ensemble and nature run are constructed by combining two data sets from the European Center for Medium‐Range Forecasts (ECMWF): the ECMWF Reanalysis Version 5 (ERA5; Hersbach et al. (2020)) and the ECMWF's 50‐member perturbed forecasts (Swinbank et al., 2016). The ERA5 data set is downloaded for every hour between 0000 UTC on 15 October to 1800 UTC on 18 October from the ECMWF's Climate Data Store (CDS). The ECMWF's perturbed forecasts are produced as part of The Observing System Research and Predictability Experiment Interactive Grand Global Ensemble (TIGGE; Swinbank et al. (2016)) and is downloaded for 0000 UTC on 15 October from the ECMWF's Meteorological Archival and Retrieval System (MARS).

The ERA5 and ECMWF's 50‐member perturbed forecasts (TIGGE ensemble) are processed using the WRF Preprocessing System and WRF's real data processor (real.exe) to produce a set of 51 WRF initial conditions files. Note that the ERA5 is used to fill in the data missing from the TIGGE ensemble above 200 hPa. The 50 WRF initial conditions from the TIGGE ensemble are then recentered on the ERA5 WRF initial condition file. The end result is a 51‐member ensemble of WRF initial conditions, where member 51 is based entirely on the ERA5 (i.e., the 51st ensemble perturbation is zero). Note that this 51st member is not used to initialize the nature run. One of the other initial conditions is used to initialize the nature run.

The lower and lateral boundary conditions used in this study are based entirely on the hourly ERA5 data set (i.e., the boundary conditions are unperturbed). While perturbed boundary conditions can increase the ensemble spread, the ensemble spread is usually reasonable even with unperturbed boundary conditions (not shown). Furthermore, as a first approach to studying the potential impacts of the BGEnKF in a high‐order weather model setting, we want the differences between the nature run (described later) and the OSSE ensemble to be entirely due to differences in the initial conditions. Future work can extend this study to situations with perturbed boundary conditions.

We desire a nature run that is roughly one ensemble standard deviation from our experiments' ensembles. To select an appropriate initial condition file for such a nature run, we first integrate the 51 members forward for 12 hr (from 0000 UTC to 1200 UTC on 15 October 2011). This integration is performed to generate flow‐dependent ensemble statistics that are consistent with the WRF model. After the 12‐hr integration, we compute the following perturbation length metric (*D* ^2^) for each of the 51 ensemble members

(9) $$D^{2}\left(n\right)\equiv \frac{1}{N_{S}\;N_{i}\;N_{j}}\sum_{v\in S}\sum_{i=1}^{N_{i}}\sum_{j=1}^{N_{j}}{\left(\frac{\Lambda \left(i,j,v,n\right)-{\langle \Lambda \left(i,j,v\right)\rangle }_{n}}{\sigma_{i,j,v}}\right)}^{2}.$$

$\Lambda \left(i,j,v,n\right)$ here is the value of a WRF‐derived field *v* at horizontal index location (*i*, *j*) for ensemble member *n*. Furthermore, ${\langle \Lambda \left(i,j,v\right)\rangle }_{n}$ is the ensemble average of $\Lambda \left(i,j,v,n\right)$, and *σ*_*i*,*j*,*v*_ is the ensemble standard deviation of $\Lambda \left(i,j,v,n\right)$. This means that the expression in the parentheses of Equation 9 is the spread‐normalized displacement of ensemble member *n* from the ensemble mean at location (*i*, *j*) for variable field *v*. The set *S* contains three 2D variables (precipitable water, column mass, and mass‐integrated kinetic energy) and *N*_*s*_ is the size of the set *S* (i.e., *N*_*S*_ = 3). Furthermore, *N*_*i*_ (≡ 432) is the number of east‐west grid points and *N*_*j*_ (≡ 243) is the number of north‐south grid points. The metric in Equation 9 can thus be interpreted as being proportional to the spread‐normalized Euclidean length of the *n*th ensemble perturbation. As such, a *D* ^2^ value of unity means that the ensemble member is generally displaced from the ensemble mean by 1 standard deviation.

We define our nature run member to be the member whose *D* ^2^ value is closest to unity at 1200 UTC on 15 October. As a result, the nature run is based on member 10 of the TIGGE ensemble. The remaining 50 WRF members will be used for our cycling OSSE DA experiments.

### 3.4 Sanity Check of Nature Run

Before proceeding, the nature run is checked by comparing it against the MERG data set. Figures 2b and 2c show longitude‐time diagrams of the Window‐BT percentiles from the MERG data set and our nature run. The construction of these percentiles is explained in Section 3.1 and in the caption of Figure 2.

We have opted to display the Window‐BT percentiles instead of the Window‐BT values because the WRF model tends to under produce clouds (i.e., when compared to satellite observations, the nature run Window‐BTs are warm biased). This is illustrated by the dashed contours in Figures 2b and 2c, which highlights areas where the latitudinally averaged values of Window‐BT were cooler than 260 K. These areas are substantially larger in the MERG data than in the nature run, meaning that the nature run under produced clouds. More specifically, the nature run over‐produces clear model columns, and under‐produces trade cumuli, cumulus congesti and stratocumuli. These likely result from imperfections in microphysics schemes and our WRF model's coarse horizontal resolution (e.g., Bryan and Morrison (2012)). Since converting the Window‐BT values to percentile values weakens the visual interference from the cloud biases, we have opted to display the Window‐BT percentiles over the Window‐BT values.

Figure 2c indicates that the nature run also exhibits the two persistent convective regions observed in the MERG data set (see Section 3.1). These persistent convective regions are indicated by the blue rectangle and blue oval in Figure 2c. Not only did the nature run's two persistent convective regions occur in locations and times similar to those of the MERG data set (Figure 2b), these nature run regions also exhibit westward propagation patterns similar to those of the MERG data set. As such, the nature run simulation reasonably replicates the anomalous convective behavior of the real atmosphere between 15 October and 18 October 2011.

### 3.5 Setup of DA Experiments to Test the BGEnKF

To test the BGEnKF, three 50‐member ensemble experiments are conducted. All three experiments start at 1200 UTC on 15 October and terminate at 1200 UTC on 18 October, with hourly DA cycling (73 cycles in total). The construction and spin‐up of these 50 members are described in Section 3.3.

In the first experiment, no observations are assimilated (henceforth, NoDA experiment). The NoDA experiment serves as a baseline for comparing the performance of the EnKF and BGEnKF, and to measure imbalances induced by DA.

The other two experiments are the EnKF and BGEnKF experiments. The only difference between the EnKF and BGEnKF experiments is in the DA algorithm employed. The EnKF experiment will assimilate observations using the PSU‐EnKF's (Meng & Zhang, 2007, 2008) default EnKF algorithm, and the BGEnKF experiment will assimilate observations using a new implementation of the BGEnKF into the PSU‐EnKF. Note that both the EnKF and the BGEnKF are implemented into the PSU‐EnKF using the high‐latency strategy proposed by Anderson and Collins (2007). The variables updated by the PSU‐EnKF include the three‐dimensional wind vector fields, potential temperature, pressure, geopotential, specific humidity, cloud liquid mass mixing ratio, rain mass mixing ratio, cloud ice mass mixing ratio, snow mass mixing ratio and graupel mass mixing ratio.

As a first approach to testing the BGEnKF, only synthetic *Meteorological Satellite 7* Meteosat Visible Infra‐Red Imager (MVIRI) Window‐BT observations will be assimilated. The MVIRI window channel has a central wavelength of 11.5 microns. Under clear sky conditions in the Tropics, the Window‐BTs are sensitive to the land/ocean surface temperature as well as the low‐to‐middle tropospheric specific humidity and temperature (Cooperative Institute for Meteorological Satellite Studies, 2022; Schmit et al., 2017). Aside from that, we will use synthetic MVIRI water vapor channel brightness temperatures (WV‐BT; central wavelength of 6.4 microns) for validation. Future work can investigate if our findings can be extended to situations where an entire suite of operationally assimilated observations and observations from different infrared channels are assimilated.

Note that we have chosen to assimilate Window‐BTs over the brightness temperatures of other infrared channels for two reasons. First, all past and present geostationary satellite infrared imagers have a window channel and an upper troposphere water vapor infrared channel. To ensure that our results can be generalized to all past and present geostationary satellite infrared imagers, our selection is limited to these two infrared channels. Second, the Window‐BT forecast statistics are generally more non‐Gaussian than those of WV‐BT. We have thus chosen to assimilate Window‐BTs over WV‐BTs because Window‐BTs will cause worse violations of the EnKF's Gaussian forecast assumption than WV‐BTs. Whether the BGEnKF is better than the EnKF at assimilating the brightness temperatures of other infrared channels is a question that deserves future investigation.

The synthetic Window‐BT observations are constructed by first running the Community Radiative Transfer Model (CRTM; Han et al. (2006)) release 2.3.0 on the nature run (see Sections 3.3 and 3.4). The CRTM simulates Window‐BT by computing the infrared window waveband radiation emitted, absorbed and scattered by all atmospheric gaseous constituents, hydrometeors, and land/ocean surface. To do so, the atmospheric temperature, specific humidity and hydrometeor mixing ratio profiles, surface temperature, and surface type information from the nature run are ingested by the CRTM. The CRTM accounts for the spectral response function of the MVIRI infrared window channel and does not use any prescribed weighting function. In other words, the CRTM explicitly solves the radiative transfer equations to generate the synthetic Window‐BT values.

The nature run's Window‐BT values are then thinned to a horizontal spacing of 27‐km (∼11,500 observations per DA cycle). To be precise, we subset the Window‐BT values by selecting values at every three grid points in the east‐west and north‐south directions. The thinned Window‐BT values are thus arranged on a regular grid with a grid spacing of 27‐km. Note that the super‐observation strategy (e.g., F. Zhang et al. (2004)) is not used in this study.

White noise with a standard deviation of 3 K is then added to the thinned nature run Window‐BT values to simulate instrument noise, thus constructing the synthetic observations. Note that the observation errors are likely to be correlated in reality. This means our use of white noise is an imperfect approximation to actual observation errors. Future work can investigate if our results can be extended to situations with correlated Window‐BT observation errors.

Common heuristic strategies are employed to assimilate the Window‐BT observations. To limit the impact of sampling errors, horizontal localization is applied using the Gaspari‐Cohn fifth‐order polynomial (Gaspari & Cohn, 1999) with a 100‐km radius of influence (Greybush et al., 2011; P. L. Houtekamer & Mitchell, 2001; P. L. Houtekamer & Zhang, 2016). No vertical localization is employed (i.e., the vertical radius of influence is infinite). Future work can seek more optimal localization settings and investigate the possibility of using adaptive localization strategies (e.g., Anderson (2012), Lei and Anderson (2014), and Lei et al. (2016, 2020)).

We also employ the Adaptive Observation Error Inflation scheme of Minamide and Zhang (2017) to limit the deleterious increments that can result from clear‐cloudy disagreements between the prior and observations (Minamide & Zhang, 2017; F. Zhang et al., 2016). To mitigate the tendency for ensemble under‐dispersion to occur when the ensemble is clear and the observation is cloudy, the Adaptive Background Error Inflation scheme of Minamide and Zhang (2019) is applied. We also employ 80% relaxation to prior perturbations to maintain ensemble dispersion (F. Zhang et al., 2004). Similar combinations of heuristic strategies are commonly seen in the EnKF‐based DA of infrared radiance observations (Chan & Chen, 2022; Chan, Zhang, et al., 2020; Minamide & Zhang, 2018; F. Zhang et al., 2016; Y. Zhang et al., 2019; Y. Zhang et al., 2021).

Aside from these common strategies, we also restrict the BGEnKF/EnKF from updating the domain‐averaged specific humidity (QVAPOR) using Window‐BT observations. Without this measure, both the BGEnKF and the EnKF experience filter divergence that is related to DA‐induced dry biases within 48 hr of cycling. These dry biases are likely induced by the ensemble's tendency to be overly cloudy. The dry biases in the EnKF experiment are likely partly because of the EnKF's inability to handle clear and cloudy members separately (see Section 4.3). As for the BGEnKF experiment, the dry bias can be explained by the fact that the BGEnKF algorithm frequently switches over to the EnKF algorithm (see Section 4.2). Note that the BGEnKF generated smaller dry biases than the EnKF (not shown).

To prevent filter divergence due to DA‐induced dry biases, we replace the 3D posterior mean QVAPOR field $\left(\bar{q_{v}^{a}}\right)$ with the following modified mean QVAPOR field $\left(\bar{q_{v}^{*}}\right)$:

(10) $$\bar{q_{v}^{*}\left(i,j,k\right)}\equiv \bar{q^{a}\left(i,j,k\right)}-\frac{1}{N_{i}\;N_{j}}\sum_{i=1}^{N_{i}}\sum_{j=1}^{N_{j}}\left\{\bar{q_{v}^{a}\left(i,j,k\right)}-\bar{q_{v}^{f}\left(i,j,k\right)}\right\}.$$

Here, (*i*, *j*, *k*) refer to the west‐east, south‐north and bottom‐top indices of the 3D QVAPOR fields and $\bar{q_{v}^{f}}$ refers to the 3D prior mean QVAPOR field.

### 3.6 Execution Wall‐Time of the BGEnKF

Before proceeding, we should compare the execution wall‐time of the BGEnKF and the EnKF. The BGEnKF algorithm took ∼30 s to assimilate ∼11,500 observations using 228 Intel Knight's Landing computer cores [distributed across 7 computational nodes on the National Energy Research Scientific Computing Center (NERSC) Cori supercomputer; each core has a clock rate of 1.4 GHz]. Assimilating the same observations via an EnKF algorithm took ∼20 s of wall‐time. For a fair comparison, this EnKF algorithm used the exact same code structure and computing resources, but with the cluster transfer and auxiliary variable update steps disabled. In other words, the BGEnKF used ∼10 s more wall‐time than the EnKF.

This ∼10‐s difference should be assessed in the context of the wall‐time for the entire PSU‐EnKF executable. The other components of the PSU‐EnKF took ∼100 s to execute. As such, the BGEnKF only added ∼10% wall‐time to the entire PSU‐EnKF executable. The BGEnKF algorithm is thus likely affordable for research and operational groups that are already running serially assimilating EnKFs (e.g., Anderson et al. (2009)).

## 4 Perfect Model WRF OSSE Results

In the discussions to follow, we will be showing plots of normalized root‐mean‐square errors (nRMSEs) and normalized biases as functions of time and model level. The normalization is necessary for the ease of visualization, and uses the root‐mean‐square errors (RMSEs) of the NoDA experiment. The EnKF experiment's nRMSE at model level *k* and date *t* is defined as

(11) $$\text{EnKF}\;\text{nRMSE}\left(k,t\right)\equiv \frac{\text{EnKF}\;\text{RMSE}\left(k,t\right)}{\text{NoDA}\;\text{RMSE}\left(k,t\right)}$$

and likewise for that of the BGEnKF and NoDA experiments (the NoDA's nRMSE values are always 1). To be clear, all of the RMSEs here are the RMSEs of the forecast ensemble mean (i.e., the NoDA RMSEs are defined as the root‐mean‐square of $\bar{\psi^{f}}-\psi^{truth}$). Note that if a filter results in nRMSEs > 1.0, the assimilation of Window‐BT via this filter degraded the ensemble with respect to the NoDA experiment. The reverse is true for nRMSEs < 1.0. We also define the normalized bias of the EnKF experiment to be

(12) $$\text{EnKF}\;\text{normalized}\;\text{bias}\left(k,t\right)\equiv \frac{\text{EnKF}\;\text{bias}\left(k,t\right)}{\text{NoDA}\;\text{RMSE}\left(k,t\right)},$$

and likewise for the BGEnKF and NoDA experiments. These biases are computed by subtracting the nature run fields from the forecast ensemble mean fields.

**Figure 3:**
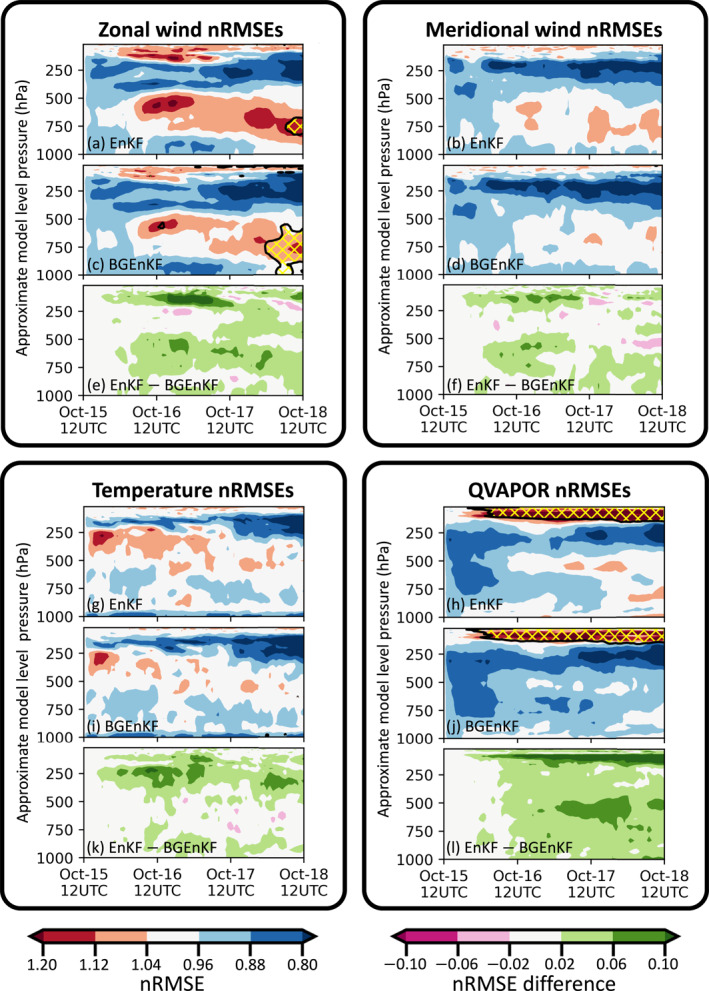
Plots of various prior ensemble statistics as functions of time and model level. For ease of interpretation, the model levels are displayed in terms of their approximate pressure levels (estimated using the definition of eta levels in WRF and assuming a surface pressure of 1,000 hPa). The shadings indicate the NoDA‐normalized RMSEs (nRMSEs; defined in Equation 11) for the EnKF (a, b, g, and h) and BGEnKF (c, d, i, and j) experiments, as well as the nRMSE differences between the EnKF and BGEnKF experiments (e, f, k, and l). The nRMSEs and nRMSE differences are shown for the U field (a, c, and e), V field (b, d, and f), T field (g, i, and k), and Q field (h, j, and l). The areas outlined with a black contour and filled with yellow hatching have consistency ratios (spread/error) less than 0.75. Note that all displayed statistics are forecast statistics.

**Figure 4:**
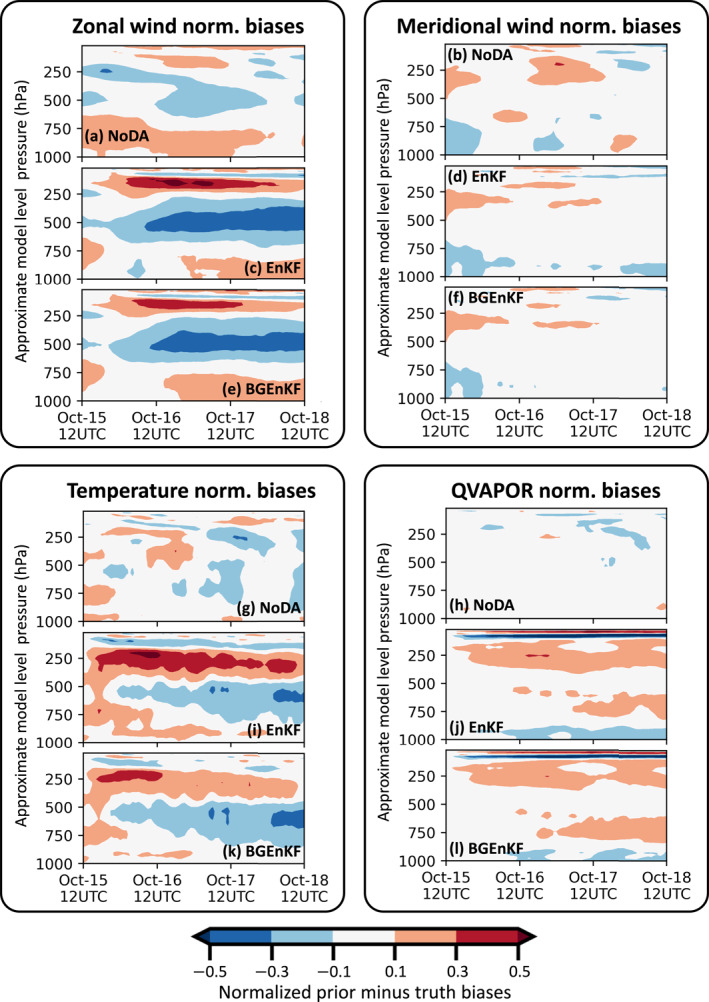
Plots of various prior ensemble normalized biases as functions of time and model level. These normalized biases are displayed for the U field (a, c, and e), V field (b, d, and f), T field (g, i, and k), and Q field (h, j, and l), for the NoDA (a, b, g, and h), EnKF (c, d, i, and j) and BGEnKF (e, f, k, and l) experiments. Similar to Figure 3, the model levels are displayed in terms of approximate pressure levels. See Equation 12 for the definition of the normalized biases.

The nRMSEs and normalized biases are examined for six variable fields: the zonal wind velocity component field (U), the meridional wind velocity component field (V), the temperature field (T), the QVAPOR field (Q), the Window‐BT field, and the upper tropospheric infrared water vapor channel brightness temperature field (WV‐BT; central wavelength of 6.2 μm). The nRMSEs are plotted in Figures 3, 6, and 6b and the normalized biases are plotted in Figures 4, 6, and 6d. All quantities are computed using forecast statistics (i.e., computed using first guesses).

### 4.1 On Differences in the BGEnKF's and the EnKF's Performances During DA Cycling

**Figure 5:**
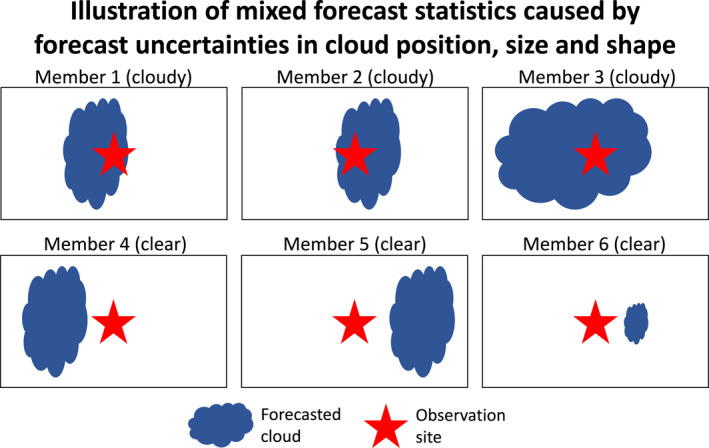
An illustration showing 6 forecast members that disagree on the location, size and shape of a forecasted cloud. As a result of these disagreements, some members are clear at the observation site, and others are cloudy at the same site.

**Figure 6:**
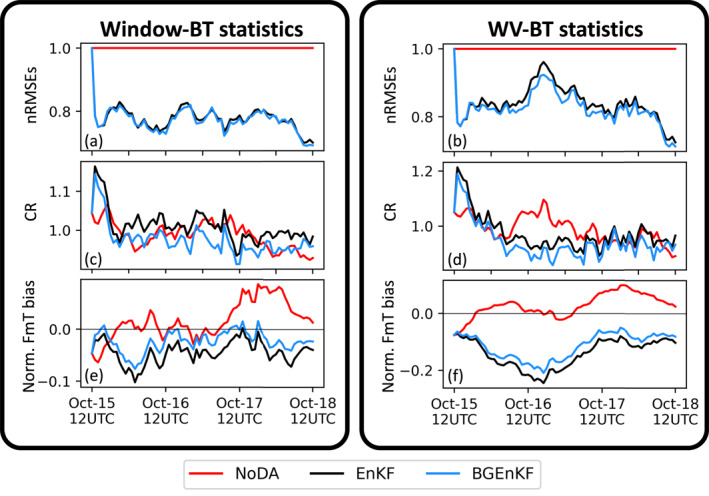
Time‐series showing the performance statistics of the three experiments' prior ensembles in terms of Window‐BT (a, c, and e) and WV‐BT (b, d, and f). The definitions of nRMSEs (a) and (b) and normalized prior minus truth (Norm. FmT bias; (e) and (f)) are the same as in Figures 3 and 4. The consistency ratio (CR; (c) and (d)) here is defined as the ratio of spread to error.

The nRMSEs and normalized biases of the BGEnKF experiment are generally better than or comparable to those of the EnKF experiment (Figures 3, 4, 5, 6). For the U, V, T, and Q fields, subtracting the BGEnKF's nRMSEs from the EnKF's nRMSEs generally results in positive values (Figures 3e, 3f, 3k, and 3l). The BGEnKF experiment also has better WV‐BT nRMSEs than the EnKF experiment (Figure 6b). The BGEnKF experiment also has smaller biases than the EnKF experiment in several places: the 100 hPa U field (Figures 4c and 4e), the 400–100 hPa T field (Figures 4i and 4k), the Window‐BT field (Figure 6e), and WV‐BT field (Figure 6f). Otherwise, the BGEnKF and EnKF experiments have similar bias values. These results suggest that the BGEnKF is more suitable for assimilating all‐sky Window‐BT than the EnKF.

The BGEnKF's performance advantages over the EnKF can be separated into two types. In the first type, the BGEnKF generates larger improvements than the EnKF (i.e., BGEnKF nRMSEs < EnKF nRMSEs < NoDA nRMSEs). This type of performance advantage occurs in multiple places (Figures 3 and 6): (a) the 800–1,000 hPa U field nRMSEs during the first 56 cycles, (b) the 100–500 hPa U field nRMSEs during the last 36 DA cycles, (c) the near surface and ∼250 hPa V field nRMSEs from 0000 UTC on 16th October to 0000 UTC on 17th October, (d) between 100 and 300 hPa in the T field nRMSEs for most cycles, (e) between 250 and 600 hPa in the Q field nRMSEs for most cycles, and in the WV‐BT nRMSEs for most DA cycles after 0000 UTC on 16th October. These differences are likely due to the BGEnKF's ability to handle mixture statistics, and suggest that the BGEnKF is more suitable for assimilating Window‐BT than the EnKF.

The BGEnKF likely produces larger improvements than the EnKF in situations where the forecast ensemble is uncertain about the absence/presence of clouds. One situation where this uncertainty likely happens is when the forecast members disagree on the location, size and/or shape of the cloud. An example of this situation is illustrated in Figure 5. As a result of these disagreements, some forecast members can be cloudy over the observation site (e.g., members 1, 2, and 3 in Figure 5) and the remaining forecast members can be clear over the same site (e.g., members 4, 5, and 6 in Figure 5). Since the BGEnKF is designed to handle mixed forecast statistics and the EnKF cannot handle such statistics, the BGEnKF produces stronger improvements than the EnKF in such a situation.

The BGEnKF experiment's second type of performance advantage over the EnKF experiment is when the BGEnKF introduces milder degradations than the EnKF (i.e., NoDA nRMSEs < BGEnKF nRMSEs < EnKF nRMSEs). In terms of nRMSEs (Figure 3), such situations are noticeable at the 100 hPa tropopause level and 500–700 hPa levels for the U and V fields, at the 200–500 hPa model levels for the T field, and at the 100 hPa level for the Q field. Such situations are also noticeable in the normalized biases of the ∼100 hPa U field, the 100–400 hPa T field (Figure 4), and in the Window‐BT and WV‐BT fields (Figure 6). These are likely because (a) the BGEnKF can handle mixture statistics whereas the EnKF cannot, and (b) the BGEnKF experiment has smaller increments than the EnKF experiment because the BGEnKF experiment has smaller dispersion. Figures 3a and 3c show an example of the latter: the BGEnKF U field has larger areas of low spread‐to‐error ratios (<0.75) than the EnKF. The likely origins of the RMSE and bias degradations are discussed in Section 4.2. Nonetheless, these results further support the notion that the BGEnKF is more appropriate for assimilating Window‐BT observations than the EnKF.

The BGEnKF tends to result in smaller spread‐to‐error ratios than the EnKF because the BGEnKF can outright convert all clear member columns to cloudy member columns, or vice versa. Since clear and cloudy member columns are very different, having both types of columns present at the same time boosts the ensemble spread. If all clear member columns are converted to cloudy member columns, or vice versa, large perturbations relative to the ensemble mean are replaced with smaller perturbations. This replacement results in reduced ensemble dispersion. Since the EnKF lacks this mechanism of ensemble spread removal, the BGEnKF can remove more ensemble spread than the EnKF, thus resulting in smaller CRs than the EnKF. Future work can investigate if stronger inflation schemes are more appropriate for the BGEnKF.

Note that there are occasional situations where the EnKF outperforms the BGEnKF. For instance, at around 0000 UTC on 17th October the BGEnKF's U nRMSEs are slightly higher than the EnKF at 250 hPa (Figure 3e). Other examples include the T nRMSEs around 1200 UTC on 17th October (Figure 3). Nonetheless, if we integrate the forecast ensembles' nRMSEs with respect to pressure at every cycle, the resulting mass‐weighted nRMSEs of the BGEnKF experiment will be lower than those of the EnKF experiment.

We have also examined day‐long deterministic forecasts that are initialized from the analysis means of the EnKF and BGEnKF experiments (not shown). The BGEnKF experiment's RMSE performance advantage over the EnKF experiment persists for up to 9 hr of lead time in terms of the U, V, and T fields. In terms of the 500–800 hPa Q field RMSEs, the BGEnKF experiment's RMSE advantage over the EnKF experiment persists throughout the 24 hr of integration. These results are as expected since the BGEnKF experiment has lower RMSEs than the EnKF experiment during DA cycling.

### 4.2 On the Similar Patterns Observed in the Performances of the BGEnKF and EnKF Experiments

**Figure 7:**
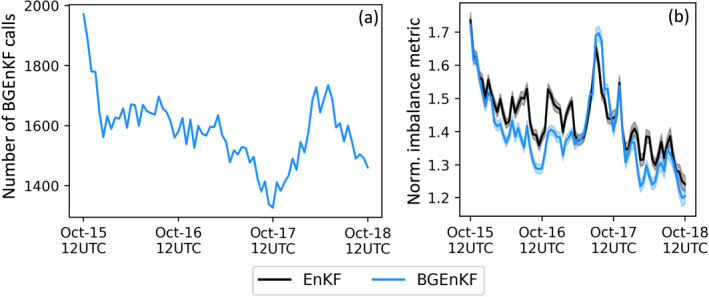
Plots showing the frequencies at which the two kernel bi‐Gaussian extension of the ensemble Kalman filter (BGEnKF) update procedure is called in the BGEnKF experiment (a), and the normalized imbalance metric statistics for both the BGEnKF and ensemble Kalman filter experiments (b). The frequency of the BGEnKF calls are plotted in terms of percentage with respect to the total number of IR observations (11,502). For example, 20% BGEnKF calls means that 20% of the IR observations are assimilated using the two kernel BGEnKF update procedure. The normalized imbalance metric is defined in the text. The solid curves in (b) indicate the ensemble average of every member's normalized imbalance metric and the half‐width of the shadings in (b) indicate twice the standard error of the members' normalized imbalance metric.

Though the BGEnKF experiment generally outperformed the EnKF experiment, there are common spatiotemporal patterns in their nRMSEs and normalized biases. For instance, Window‐BT DA with either algorithm tends to degrade the 500–800 hPa U nRMSEs, and improve the 100–500 hPa U nRMSEs (Figures 3a and 3c). These similarities are likely because the BGEnKF frequently switches over to the EnKF. Figure 7a shows that the BGEnKF algorithm is only called to assimilate ∼10% of the Window‐BT observations, meaning that the switching occurred for the remaining ∼90% of Window‐BT observations. Future work can investigate if reducing the occurrence of such switches (e.g., via weaker heuristic checks and larger ensembles) could improve the performance of the BGEnKF.

It is notable that the BGEnKF outperforms the EnKF despite the high frequency of BGEnKF‐to‐EnKF switching. For instance, according to Figures 3h, 3j, and 3l, for the 24 cycles on 17th October and between 500 and 700 hPa, the BGEnKF experiment has 0.06–0.1 less Q nRMSEs than the EnKF experiment. Since the EnKF experiment has Q nRMSEs of ∼1 then, the BGEnKF is able to introduce a ∼6%–10% improvement over the EnKF. These are considerable improvements since the BGEnKF is only called on ∼10% of the Window‐BT observations.

Given the frequent switching from the BGEnKF to the EnKF, the worse‐than‐NoDA RMSEs and biases in both the EnKF and BGEnKF experiments are likely caused by the EnKF algorithm. These degradations are likely caused by (a) non‐Gaussian forecast statistics, (b) sampling errors, and (c) biases that are introduced by the assimilation of Window‐BT. The first factor originates from having mixtures of clear and cloudy members. Earlier work has demonstrated the existence of the non‐Gaussian forecast statistics in the brightness temperature fields (Chan, Anderson, & Chen, 2020; Harnisch et al., 2016; Minamide & Zhang, 2017) and we have furnished additional evidence in Text S1 of the Supporting Information S1. Sampling errors can also introduce errors into the analysis, particularly over regions where the ensemble correlations are weak. This factor is likely present in our experiments because no vertical localization is used in this study. Future work can investigate if vertical localization can mitigate some of the RMSE and bias degradations (Lei & Anderson, 2014; Lei & Whitaker, 2015; Lei et al., 2016, 2020). Finally, since biases are a component of RMSEs (e.g., Ying and Zhang (2017), Ying and Zhang (2018), and Chan, Zhang, et al. (2020)), biases that are introduced by Window‐BT DA can contribute toward worse‐than‐NoDA RMSEs. While the contribution of biases to worse‐than‐NoDA RMSEs can be easily inferred (see the next paragraphs), the contributions from the first two factors cannot be easily teased apart.

To understand the contribution of biases to the occurrence of worse‐than‐NoDA RMSEs (i.e., nRMSEs > 1), we computed the following fraction as a function of model level and time $\left(f_{\text{bias}}\left(k,t\right)\right)$. For the EnKF experiment, we defined

(13) $${\text{EnKF}}^{\prime }s\;f_{\text{bias}}\left(k,t\right)\equiv \sqrt{\frac{{\left[{\text{EnKF}}^{’}s\;\text{biases}\left(k,t\right)\right]}^{2}-{\left[{\text{NoDA}}^{’}s\;\text{biases}\left(k,t\right)\right]}^{2}}{{\left[{\text{EnKF}}^{’}s\;\text{RMSEs}\left(k,t\right)\right]}^{2}-{\left[{\text{NoDA}}^{’}s\;\text{RMSEs}\left(k,t\right)\right]}^{2}}}$$

and likewise for the BGEnKF experiment. *f*_bias_ can be interpreted as the fractional contribution of biases to the worse‐than‐NoDA RMSE performance.

We found that for about 25%–45% of the worse‐than‐NoDA situations (nRMSEs > 1) in the U and T fields, the majority of the nRMSE degradation (i.e., *f*_bias_ ≥ 0.6) can be explained by the introduction of biases [i.e., $p\left(f_{\text{bias}}>0.6\;|\text{nRMSE}>1\right)\in \left(0.25,0.45\right)$]. This suggests that though DA‐induced biases are important contributors toward the worse‐than‐NoDA RMSEs of either DA filters, the net contribution coming from other factors is also important. Future work can examine separating and quantifying the relative importance of these three factors toward the worse‐than‐NoDA RMSEs.

### 4.3 On the Origin of Biases in the EnKF and BGEnKF Experiments

We now turn our attention to the U, T, Q, Window‐BT, and WV‐BT biases that are introduced by Window‐BT DA. Since the Q analysis increments are subject to bias removal (see last paragraph of Section 3.5), the Q biases will be discussed later. The U, T, and WV‐BT biases are likely related to (a) a cold forecast minus truth (FmT) Window‐BT bias at the start of all experiments, and (b) the persistence of these FmT Window‐BT biases throughout all cycles (Figure 6e). Item 1 is essentially the result of drawing a single member from an ensemble—it is difficult to obtain a nature run whose domain‐averaged Window‐BT is always the same as that of the forecast ensemble. This is supported by the fact that the NoDA experiment's FmT Window‐BT biases oscillate around zero (Figure 6e). More interestingly, item 2 indicates an over abundance of clouds in both DA experiments. Since WV‐BT is cooler in the presence of clouds, the WV‐BT bias is explained by the over abundance of clouds.

To understand the origin of the persistently cold FmT Window‐BT biases, we examine the analysis ensembles' Window‐BT biases. Running the CRTM on the analysis ensembles of the Window‐BT DA experiments reveals analysis minus truth (AmT) Window‐BT normalized biases that are typically around −0.25 (not shown). These bias values are a factor of 5 larger than the FmT normalized biases of around −0.05 (Figure 6e). The large AmT biases suggest that Window‐BT DA resulted in overly cloudy analysis ensembles. Though the time‐integration of these analysis ensembles dramatically reduces the over cloudiness (the normalized biases typically go from −0.25 to −0.05), some over cloudiness likely remain. As such, the U, T, Window‐BT, and WV‐BT biases are likely caused by the EnKF and BGEnKF experiments introducing too many clouds into the analysis ensemble.

The over introduction of clouds is likely a result of the EnKF's inability to handle clear and cloudy members separately and the strong sensitivity of Window‐BTs to hydrometeors. When both clear and cloudy members are present in the forecast ensemble, the EnKF's forecast mean state will contain some amount of clouds. Suppose that the correlations between Window‐BT and hydrometeor mixing ratios are negative. If Window‐BT observations with either small or negative innovations are assimilated, the clouds in the EnKF's mean state will either be unaffected (for small innovations) or be increased (for negative innovations). Since the EnKF will also reduce the size of the ensemble members' perturbations, the ensemble thus contracts around a cloudy mean state. The result is that clear column forecast members gain some amount of clouds, even in situations where the innovations are close to zero. Since Window‐BTs are sensitive to the presence of clouds, running the CRTM on such members will generate cold cloudy Window‐BT values. This mechanism of EnKF‐induced over‐cloudiness warrants future investigation.

Note that the BGEnKF experiment's over‐cloudiness is likely caused by the mechanism in the previous paragraph. This is because the BGEnKF algorithm frequently switches over to the EnKF (for ∼90% of assimilated observations). Since the BGEnKF can handle mixtures of clear and cloudy members, with less frequent switches, the BGEnKF is likely to have smaller biases. To test this possibility, smaller sampling errors are necessary to justify less frequent switches from the BGEnKF to the EnKF. Future work can thus investigate this possibility with larger ensembles.

With regards to the Q biases, since the analysis increment cannot modify the Q biases (see Equation 10), these biases are induced during the forecast step of the DA procedure. We can rule out the evaporation of DA‐induced spurious clouds as an important source because the hydrometeor biases injected by the increment are an order of magnitude smaller than the Q bias growth during integration (not shown). Other processes are likely causing the Q biases. Some possibilities include enhancements to the upward transport of Q from the surface and/or the latent fluxes from the ocean surface. The exact origin of these Q biases can be investigated in future work.

### 4.4 On Dynamical Imbalances

Note that the BGEnKF introduces less dynamical imbalances into the ensemble than the EnKF. To measure dynamical imbalance, we compute the root‐mean‐square of the second time derivative of surface pressure during the time integration phase of each DA cycle (P. Houtekamer & Mitchell, 2005; Temperton & Williamson, 1981). These derivatives are computed via centered differencing (Press & Flannery, 2010) on three consecutive snapshots of the surface pressure field. These snapshots are spaced 30‐min apart. The resulting imbalance metric is normalized using the NoDA experiment's imbalance metric. A normalized imbalance metric value of 1 indicates that a normal amount of fast‐moving gravity waves is present. A value greater than 1 indicates that a higher than normal amount of fast‐moving gravity waves is present, thus indicating DA‐induced imbalances.

Figure 7b indicates that the BGEnKF experiment generally has either statistically indistinguishable or milder imbalances than the EnKF experiment. The only exception to this trend happens between 0000 UTC to 1200 UTC on 17th October. The BGEnKF is thus likely more appropriate than the EnKF at assimilating Window‐BT observations.

This difference in imbalance is likely due to two factors. First, because the BGEnKF is better at treating non‐Gaussian forecast statistics than the EnKF, the BGEnKF algorithm's increments are likely more balanced than those of the EnKF algorithm. Second, because the BGEnKF experiment has generally less dispersion than the EnKF experiment, the BGEnKF experiment's increments are likely smaller than those of the EnKF experiment. Future work can investigate the relative contributions of these two factors to the BGEnKF's milder‐than‐EnKF imbalance.

### 4.5 On the Relationship Between the Frequency of BGEnKF Calls and Filter Performance

We have also examined how the performance of the BGEnKF experiment varies with the frequency of BGEnKF calls. This relationship is measured using the Spearman rank correlations between (a) the RMSE changes that are induced by the DA increment (ΔRMSEs), and (b) the frequency of BGEnKF calls. To be clear, we have defined

(14) ΔRMSE≡nRMSEofposteriormean−nRMSEofpriormean.

Both positive and negative statistically significant Spearman rank correlations (*p*‐value < 0.1) are observed, meaning that two types of statistical relationships are detected. First, there are statistically significant negative Spearman rank correlations between the frequency of BGEnKF calls and the ΔRMSEs of the lower tropospheric V, mid‐tropospheric and near‐surface T, and ∼700 hPa QVAPOR. In other words, more frequent BGEnKF calls is correlated with improvements to the performance of these model quantities. The second relationship is indicated by statistically significant positive Spearman rank correlations between the frequency of BGEnKF calls and upper tropospheric ΔRMSEs for U, V, T, QVAPOR. These positive relationships imply that increases in the frequency of BGEnKF calls are correlated with worse filter performance for these upper tropospheric quantities.

A plausible explanation for these correlations is that, even when the ensemble is mixed and the BGEnKF is called, the forecast statistics at some model levels are better approximated by a Gaussian distribution than a bi‐Gaussian distribution (henceforth, the more Gaussian levels). At levels where the bi‐Gaussian forecast assumption is more appropriate than the Gaussian forecast assumption (henceforth, the more bi‐Gaussian levels), more BGEnKF calls will lead to more negative ΔRMSEs. At the more Gaussian levels, more BGEnKF calls will lead to more positive ΔRMSEs. This explanation suggests that, when the BGEnKF is called, (a) the forecast statistics of the lower tropospheric V, mid‐tropospheric and near‐surface T, and ∼700 hPa QVAPOR are more bi‐Gaussian than Gaussian, and (b) the forecast statistics of the upper tropospheric U, V, T, QVAPOR are more Gaussian than bi‐Gaussian. Note that this explanation assumes that variations in the ratio of BGEnKF calls do cause changes in ΔRMSEs. Future work can test this hypothesized explanation by measuring the deviations of forecast statistics at each model level and grid point from a bi‐Gaussian distribution and a Gaussian distribution.

## 5 Conclusions and Future Work

In this study, we compare the BGEnKF against the EnKF using perfect model OSSEs with a realistic weather model (WRF) for a case of tropical convection. These OSSEs are executed using the state‐of‐the‐art PSU‐EnKF system. Our results indicate that the BGEnKF outperforms the EnKF at assimilating synthetic Window‐BT observations. We observe this performance advantage in terms of the RMSEs and biases of the U, V, T, Q, Window‐BT, and WV‐BT fields. This performance advantage is likely due to the BGEnKF's ability to handle mixtures of clear and cloudy column members. These performance advantages are achieved even though the BGEnKF only activated for ∼10% of the assimilated Window‐BT observations. As such, these promising results motivate future work into the BGEnKF using real data.

There are several large areas of future research for the BGEnKF. The first large area concerns refining the BGEnKF algorithm. Future work can, for instance, seek less heuristic approaches to sort the ensemble into clusters in a computationally efficient manner. One option is to combine clustering algorithms (e.g., k‐means (Forgy, 1965; Lloyd, 1982), support‐vector machines (Cortes & Vapnik, 1995) and EM (Sondergaard & Lermusiaux, 2013a)) with dimension reduction methods (e.g., Sondergaard and Lermusiaux (2013a), Reddy et al. (2020), and Albarakati et al. (2021)). Since cluster sizes, and thus sampling errors, can vary in each iteration of the serial BGEnKF loop, future work can investigate using adaptive or empirical localization methods (Anderson, 2012; Anderson & Lei, 2013; Lei & Anderson, 2014) to improve the BGEnKF's performance. Future work can also examine more sophisticated methods to regulate when the BGEnKF switches over to the EnKF (e.g., using the Shapiro‐Wilk test for normality).

Another area of future work is to hybridize the BGEnKF with other DA algorithms. Hybridization with kernel filters (Anderson & Anderson, 1999; Hoteit et al., 2008, 2012; Kotsuki et al., 2022; Liu et al., 2016; Stordal & Karlsen, 2017; Stordal et al., 2011) can be achieved by assigning the clear cluster's covariance to clear member kernels and likewise for the cloudy member kernels. Existing ensemble‐variational hybrid DA algorithms (Hamill & Snyder, 2000; Lorenc, 2003; Buehner, 2005; X. Wang et al., 2007) can also be hybridized with the BGEnKF. For instance, the BGEnKF can replace the EnKF component of such methods. Hybridization with DA methods that employ transport methods to update ensemble members (Evensen et al., 2022; Hu & van Leeuwen, 2021; Marzouk et al., 2017; Reich, 2012; van Leeuwen, 2011) is also possible. This can provide a different method to shift members between clusters, as opposed to the current deletion‐resampling method. Finally, the BGEnKF can be potentially hybridized with ensemble DA methods that allow non‐parametric prior distributions. Such methods include particle filters (Poterjoy, 2016; Poterjoy et al., 2019; van Leeuwen, 2009, 2019; Vetra‐Carvalho et al., 2018), the quantile conserving ensemble filter (Anderson, 2022), and the rank histogram filter (Anderson, 2010, 2019, 2020).

Since we have only tested the BGEnKF in a perfect model WRF OSSE using Window‐BT observations, future work can test the BGEnKF in increasingly realistic scenarios, with other observation types, and/or in other Earth systems. For instance, since radar reflectivity observations are sensitive to the presence and absence of precipitation, the BGEnKF can potentially be better at assimilating such observations. The performance of the BGEnKF can also be compared with other popular DA algorithms in tests that assimilate the operational suite of atmospheric in situ and remote observations. Imperfect model OSSEs and real data tests can also be done. The BGEnKF can also be tested in other Earth system components.

This study is among the first to demonstrate the potential of the BGEnKF with a high‐order weather model. Our BGEnKF is computationally efficient, scalable with parallelization, and likely straightforward to implement in existing serial EnKF DA systems. These algorithmic properties and our promising results motivate future research into developing, testing and applying the BGEnKF, or similar Gaussian mixture model EnKFs, for Earth systems DA.

## Supporting information

Supporting Information S1

## Data Availability

The data and software used in this study are either publicly available or available upon request. The WRF model software can be found on the National Center for Atmospheric Research's WRF website (University Corporation for Atmospheric Research, 2016). Our WRF ensemble is constructed using the ECMWF TIGGE data archived on the MARS system (European Centre for Medium‐Range Weather Forecasts, 2016) and the ERA5 data archived on the CDS system (European Centre for Medium‐Range Weather Forecasts, 2016). The MERG data product is obtained from NASA's GES DISC (Janowiak et al., 2017). We have archived this study's experiments and a copy of the Fortran 90 BGEnKF module on the Pennsylvania State University's Data Commons (Chan, Chen, & Anderson, 2022). The Fortran 90 source code of the PSU‐EnKF system, including the implemented BGEnKF, is available upon request.
