# Supplementary material for: The Potential Benefits of Handling Mixture Statistics via a Bi‐Gaussian EnKF: Tests With All‐Sky Satellite Infrared Radiances
Source: J Adv Model Earth Syst. 2023 Feb 6;15(2):e2022MS003357. doi: 10.1029/2022MS003357 (PMC10078334; doi:10.1029/2022MS003357)
Supplement: Supplementary file 1 — Supporting Information S1 [file JAME-15-0-s001.pdf]

Supporting Information for "Potential benefits of handling mixture statistics via a bi-Gaussian EnKF: tests with all-sky satellite infrared radiances "

Man-Yau Chan[1,2], Xingchao Chen[1,2], Jeffrey L. Anderson[3]

[1]Department of Meteorology and Atmospheric Science, The Pennsylvania State University, University Park, Pennsylvania, USA

[2]Center for Advanced Data Assimilation and Predictability Techniques, The Pennsylvania State University, University Park, Pennsylvania, USA

[3]Data Assimilation Research Section, Computational Information Systems Laboratory, National Center for Atmospheric Research, Boulder, Colorado, USA

Contents of this file

1. Text S1: Some differences between clear and cloudy member statistics
2. Text S2: Discussion on heuristic clustering
3. Text S3: Bayes' rule for the BGEnKF
4. Text S4: A detailed description of the BGEnKF algorithm
5. Text S5: Outline of the BGEnKF algorithm serial filtering workflow
6. Text S6: On generalizing the BGEnKF algorithm to handle more clusters
7. Figure S1: Illustration of the differences between clear and cloudy ensemble statistics
8. Figure S2: Illustration of the BGEnKF algorithm serial filtering workflow

Introduction

This document has several purposes. First, we will illustrate some differences between clear ensemble statistics and cloudy ensemble statistics. Differences like these motivate research into the BGEnKF and similar GMM-EnKFs. The second purpose is to provide a quick reference for other scientists to understand the BGEnKF, independently re-create our BGEnKF algorithm, and to support further development of the BGEnKF. To increase the accessibility of this area of research, we have written this document with graduate students in mind.

1. Text S1: Some differences between clear and cloudy member statistics

To set the stage, we plotted maps of the ensemble averaged Window-BT [Figure S1(b)] and the fraction of cloudy member columns in the ensemble [Figure S1(b)]. These ensemble quantities are constructed from the spun-up 50-member WRF ensemble described in the main text. Though the ensemble captured the general appearance of the organized convective features seen in the nature run [Figures 2(a) and S1(a)], the ensemble was uncertain about the presence/absence of clouds over much of the domain [Figure S1(b)]. This uncertainty is particularly noticeable over regions where the ensemble averaged Window-BT was between 248 K and 280 K.

Several differences between clear and cloudy member columns can be seen from Figure S1. First, the average Window-BT values of clear member columns are typically warmer than 280 K, whereas the average Window-BT values of cloudy member columns are cooler than 280 K [Figure S1(c & d)]. This difference is well known. As such, the Window-BT ensemble statistics of an ensemble of clear and cloudy member columns (henceforth, mixed ensemble) will exhibit mixed statistics.

The clear and cloudy member columns also differ noticeably in terms of their humidity fields and the Kalman gain linking Window-BT innovations to humidity increments. For the ease of

visualization, we examined through a column-integrated measure of humidity that is a linear function of the WRF model state: the pseudo precipitable water (PPW). The PPW is defined as

$$\mathrm{PPW} \equiv \frac{g}{P_{\mathrm{sfc}} - P_{\mathrm{top}}} \int_0^1 q_v d\eta \tag{1}$$

where q_v refers to water vapor mass mixing ratio (QVAPOR), P_{sfc} and P_{top} refer to model surface pressure and model top pressure, and η refers to the WRF model's vertical coordinate. The PPW can be derived from the definition of precipitable water by applying the hydrostatic approximation, the definition of WRF η levels, and by assuming that P_{sfc} and P_{top} are constants ($P_{\mathrm{sfc}} \equiv 1000$ hPa, $P_{\mathrm{top}} \equiv 20$ hPa).

We opted to use the linear PPW over precipitable water (PW) because PW is a nonlinear function of the model state. Thus, the Kalman gain linking PW to Window-BT within the same model column is not mathematically equivalent to taking a column-integral of the Kalman gain linking QVAPOR to Window-BT. In contrast, said mathematical equivalence holds for PPW. Looking at PPW over PW thus allows us to get an accurate sense of what the EnKF would do to QVAPOR within a model column.

Figure S1(c & d) indicates that the PPW of cloudy member columns is higher than that of clear member columns. This is because clouds require nearly saturated humidity to materialize. As such, when the ensemble is mixed, mixture statistics in the humidity fields are likely.

We also examined the component of the Kalman gains responsible for propagating Window-BT innovations to QVAPOR: the least squares linear regression coefficient linking Window-BT to QVAPOR (Anderson, 2003). For the ease of visualization, we looked at the coefficient linking Window-BT to PPW within the same column. This coefficient (β) is defined as

$$\beta \equiv \frac{\mathrm{Cov}\,(\mathrm{PPW}, \mathrm{BT})}{\mathrm{Var}\,(\mathrm{BT})}. \tag{2}$$

$\mathrm{Cov}\,(\mathrm{PPW}, \mathrm{BT})$ denotes the prior ensemble covariance between PPW and Window-BT within said model column, and $\mathrm{Var}\,(\mathrm{BT})$ denotes the prior ensemble variance of Window-BT within the same column. In the limit where Var(BT) is much smaller than the observation error, the Kalman gain turns into β.

As can be seen from Figure S1(e & f), the clear member columns' statistically significant β values are generally an order of magnitude larger than those of the cloudy member columns. This difference suggests that the statistical relationship between Window-BT and humidity can vary dramatically depending on the absence/presence of clouds.

2. Text S2: Heuristic localized clustering of ensemble members

Since a mixture of clear and cloudy members results in a mixed prior distribution, it seems appropriate to explore an ensemble DA method that explicitly treat mixture distributions. Since the EnKF has been remarkably successful at assimilating infrared radiance observations (Otkin, 2012; F. Zhang et al., 2016; Honda et al., 2018; Minamide & Zhang, 2018; Y. Zhang et al., 2018; Otkin & Potthast, 2019; F. Zhang et al., 2019; Geer et al., 2019; Chan, Zhang, et al., 2020; Jones et al., 2020; ?, ?; Hartman et al., 2021; Y. Zhang et al., 2021), we will extend the EnKF to handle clear members and cloudy members separately.

A complication in handling clear members and cloudy members separately lies in the fact every member usually contains both clear model columns and cloudy model columns. Supposing we

have $N_i * N_j$ model columns in the domain, there can exist up to $2^{N_i*N_j}$ possible spatial combinations of clear and cloudy columns in the domain. Sampling these $2^{N_i*N_j}$ combinations would require more than $2^{N_i*N_j}$ ensemble members – a likely impractical proposition. Dimensional reduction is necessary to reduce the required number of ensemble members.

A simple and natural dimensional reduction approach is to limit our clear/cloudy considerations to small regions of the domain. This dimensional reduction approach is effectively a type of spatial localization – a commonly employed heuristic method used to limit the effects of sampling errors on EnKFs (Houtekamer & Zhang, 2016). As a first attempt at employing this localization, suppose we are assimilating observations one-at-a-time (*i.e.,* serial assimilation). When assimilating the m-th observation, we will only consider model columns within 1 horizontal radius of influence (HROI) surrounding the observed column. If there are N_{loc} columns within 1 HROI of the observed column, the number of possible spatial combinations falls from $2^{N_i*N_j}$ to $2^{N_{loc}}$. For commonly used HROI values, $2^{N_{loc}} \ll 2^{N_i*N_j}$.

Though localization can dramatically reduce the number of spatial clear/cloudy combinations, $2^{N_{loc}}$ is likely greater than the number of ensemble members N_E. For instance, in the IR DA experiments of Chan, Zhang, et al. (2020) and ? (?), the HROI is approximately 11 model grid spacings (100-km HROI, 9-km grid spacing), meaning that there exist $\sim \pi * 11^2 \approx 363$ model columns within the localization zone. A typical ensemble size of ~ 50 is much less than the number of spatial combinations in this example ($\sim 2^{363}$). Another measure is necessary to further simplify the problem.

We opted to assume that there are at most two clear/cloudy spatial combinations within the localized zone. To understand the rationale, consider that localized serial EnKFs assume that all ensemble members within 1 radius of influence (ROI) of an observation to be drawn from a Gaussian distribution (Burgers et al., 1998; Whitaker & Hamill, 2002; Anderson, 2003). This is equivalent to assuming that there exists only one spatial combination within 1 ROI of said observation. Our two spatial combination assumption, though imperfect, is closer to the actual number of spatial combinations ($2^{N_{loc}}$) than the one spatial combination assumption.

We can now consider that the ensemble members are drawn from a mixture of two distributions within the localized region. The EnKF can be extended to handle this mixture distribution by replacing the EnKF's Gaussian prior assumption. Specifically, we consider that some prior members are drawn from one Gaussian distribution and the other members are drawn from a different Gaussian distribution. The prior ensemble is thus assumed to be drawn from a bi-Gaussian prior distribution. The resulting algorithm will be henceforth termed the bi-Gaussian EnKF (BGEnKF).

For the BGEnKF to work, it is necessary to separate the ensemble members into two groups (henceforth termed "clusters"). The sample statistics of each cluster will correspond to one of two Gaussian kernels. As a first approach, we will consider members that are clear at the observation site to be drawn from one Gaussian distribution (henceforth termed the “clear kernel” or “clear cluster”). The remaining members will be considered to be drawn from a different Gaussian distribution (henceforth, the “cloudy kernel” or “cloudy cluster”). More advanced clustering approaches, such as those involving machine learning (*e.g.*, support vector machines), can be considered at a later date.

3. Text S3: Bayes' rule for the BGEnKF

We will now formulate a serially assimilating BGEnKF (*i.e.*, the algorithm assimilates one observation at a time) starting from Bayes' rule and using a notation akin to that of Ide, Courtier,

Ghil, and Lorenc (1997). In our earlier study (Chan, Anderson, & Chen, 2020), the BGEnKF was formulated as a model state space filter [or, in the terminology of Anderson and Collins (2007), a sequential filter]. However, multi-process implementations of sequential filters require inter-process communications at every iteration of the serial assimilation loop. The sequential filter formulation thus does not scale well with parallelization (Anderson & Collins, 2007).

To ensure that the BGEnKF algorithm scales well with parallelization, the BGEnKF is formulated to constrain an extended state vector $\boldsymbol{\psi}$ (Anderson & Collins, 2007). $\boldsymbol{\psi}$ will contain all of the variables used in the BGEnKF. Aside from containing the model state $\boldsymbol{x}$, $\boldsymbol{\psi}$ will also contain the simulated observation values $\boldsymbol{y}$ that correspond to said model state. Furthermore, since ξ [column-integrated frozen water mass content; see main text's Eq. (1)] can be used to discriminate clear column members from cloudy column members (see main text's section 2.2), we will include ξ at every observation site into $\boldsymbol{\psi}$. The vector $\boldsymbol{\xi}$ will be used to denote the ξ values at every observation site. We can thus define

$$\boldsymbol{\psi} \equiv \begin{bmatrix} \boldsymbol{x} \\ \boldsymbol{y} \\ \boldsymbol{\xi} \end{bmatrix}. \tag{3}$$

Supposing N_x denotes the number of elements in $\boldsymbol{x}$ and N_y denotes the number of elements in $\boldsymbol{y}$ (and in $\boldsymbol{\xi}$), then $\boldsymbol{\psi}$ has $N_x + 2N_y$ elements. For the ease of writing, we will define

$$N_\psi \equiv N_x + 2N_y$$

With Eq. (3), we can construct an ensemble of forecasted $\boldsymbol{\psi}$ vectors. Supposing that we have a forecast ensemble of N_E model states $\left\{\boldsymbol{x_1^f}, \boldsymbol{x_2^f}, \dots, \boldsymbol{x_{N_E}^f}\right\}$, we can define an ensemble of N_E forecasted extended state vectors via

$$\boldsymbol{\psi_n^f} \equiv \begin{bmatrix} \boldsymbol{x_n^f} \\ \boldsymbol{h}(\boldsymbol{x_n^f}) \\ \boldsymbol{\xi}\left(\boldsymbol{x_n^f}\right) \end{bmatrix} \quad \forall \;\; n = 1, 2, \dots, N_E. \tag{4}$$

Here, $\boldsymbol{h}(\boldsymbol{x_n^f})$ represents calling the observation operator $\boldsymbol{h}$ on $\boldsymbol{x_n^f}$, and $\boldsymbol{\xi}\left(\boldsymbol{x_n^f}\right)$ represents evaluating ξ [Eq. (1) of main text] at every observation site using the information in $\boldsymbol{x_n^f}$.

Since the BGEnKF will be formulated as a serial assimilation algorithm, we can outline the essence of the algorithm by considering what happens when a single observation (y^o) is assimilated into an ensemble of forecasted $\boldsymbol{\psi}$ vectors. Like typical serially assimilating EnKF algorithms [*e.g.*, Whitaker and Hamill (2002), Anderson et al. (2009), and Meng and Zhang (2007)], the serially assimilating BGEnKF algorithm is of the form:

1. Construct an ensemble of forecasted $\boldsymbol{\psi}$ vectors (*i.e.*, $\left\{\boldsymbol{\psi}_{\boldsymbol{1}}^{\boldsymbol{f}}, \boldsymbol{\psi}_{\boldsymbol{2}}^{\boldsymbol{f}}, \ldots, \boldsymbol{\psi}_{\boldsymbol{N_E}}^{\boldsymbol{f}}\right\}$).
2. Select an unassimilated observation.
3. Divide the ensemble into the clear and cloudy clusters using the procedure described the main text's section 2.2.
4. Assimilate the selected observation using the BGEnKF to construct an ensemble of analyzed $\boldsymbol{\psi}$ vectors (*i.e.*, $\left\{\boldsymbol{\psi}_{\boldsymbol{1}}^{\boldsymbol{a}}, \boldsymbol{\psi}_{\boldsymbol{2}}^{\boldsymbol{a}}, \ldots, \boldsymbol{\psi}_{\boldsymbol{N_E}}^{\boldsymbol{a}}\right\}$)
5. If there are unassimilated observations remaining,
   (i) Overwrite the forecast ensemble with the posterior ensemble (*i.e.*, $\boldsymbol{\psi}_{\boldsymbol{n}}^{\boldsymbol{f}} \leftarrow \boldsymbol{\psi}_{\boldsymbol{n}}^{\boldsymbol{a}} \ \forall n = 1, 2, \ldots, N_E$).
   (ii) Return to step 2..
6. Exit.

We will thus formulate the BGEnKF equations by considering the assimilation of y^o into $\left\{\boldsymbol{\psi}_{\boldsymbol{1}}^{\boldsymbol{f}}, \boldsymbol{\psi}_{\boldsymbol{2}}^{\boldsymbol{f}}, \ldots, \boldsymbol{\psi}_{\boldsymbol{N_E}}^{\boldsymbol{f}}\right\}$. Supposing that the ensemble members have been sorted into the clear and cloudy clusters based on the ξ value at the observation site, the BGEnKF assumes that the prior probability density function [pdf; $p(\boldsymbol{\psi})$] can be represented by the bi-Gaussian pdf

$$p(\boldsymbol{\psi}) = w_{\text{clr}}^{f}\, \mathcal{G}\left(\boldsymbol{\psi};\ \overline{\boldsymbol{\psi}_{\text{clr}}^{\boldsymbol{f}}}, \boldsymbol{P}_{\text{clr}}^{\boldsymbol{f}}\right) + w_{\text{cld}}^{f}\, \mathcal{G}\left(\boldsymbol{\psi};\ \overline{\boldsymbol{\psi}_{\text{cld}}^{\boldsymbol{f}}}, \boldsymbol{P}_{\text{cld}}^{\boldsymbol{f}}\right). \tag{5}$$

Throughout this document, we will use the subscript "clr" to denote clear cluster quantities, and the subscript "cld" to denote cloudy cluster quantities. $\mathcal{G}\left(\boldsymbol{\psi};\ \overline{\boldsymbol{\psi}_{\text{clr}}^{\boldsymbol{f}}}, \boldsymbol{P}_{\text{clr}}^{\boldsymbol{f}}\right)$ denotes the clear cluster's Gaussian kernel with mean state $\overline{\boldsymbol{\psi}_{\text{clr}}^{\boldsymbol{f}}}$ and covariance matrix $\boldsymbol{P}_{\text{clr}}^{\boldsymbol{f}}$. Similarly, $\mathcal{G}\left(\boldsymbol{\psi};\ \overline{\boldsymbol{\psi}_{\text{cld}}^{\boldsymbol{f}}}, \boldsymbol{P}_{\text{cld}}^{\boldsymbol{f}}\right)$ denotes the cloudy cluster's Gaussian kernel with mean state $\overline{\boldsymbol{\psi}_{\text{cld}}^{\boldsymbol{f}}}$ and covariance matrix $\boldsymbol{P}_{\text{cld}}^{\boldsymbol{f}}$. In general, the Gaussian pdf for a K-dimensional state $\boldsymbol{p}$ vector with some mean $\boldsymbol{\mu}$ and covariance matrix $\boldsymbol{C}$ is defined as

$$\mathcal{G}(\boldsymbol{p};\ \ \boldsymbol{\mu}, \boldsymbol{C}) \equiv \frac{1}{\sqrt{(2\pi)^K \det(\boldsymbol{C})}} \exp\left\{-\frac{1}{2}(\boldsymbol{p}-\boldsymbol{\mu})^\top \boldsymbol{C}^{-1}(\boldsymbol{p}-\boldsymbol{\mu})\right\}.$$

The scalar quantities w_{clr}^{f} and w_{cld}^{f} are the respective weights of the clear and cloudy Gaussian kernels. Note that

$$w_{\text{clr}}^{f} + w_{\text{cld}}^{f} = 1, \quad w_{\text{clr}}^{f} \geq 0, \quad \text{and,} \quad w_{\text{cld}}^{f} \geq 0.$$

The various parameters in the prior pdf [Eq. (5)] are estimated from the clustered forecast ensemble of $\boldsymbol{\psi}$ vectors. Suppose the set S_{clr} contains the ensemble member indices of clear cluster members [*i.e.*, the index n in Eq. (4)] and the set S_{cld} contains the ensemble member indices of cloudy cluster members. We first compute the number of members in the clear cluster (N_{clr}^{f}) and the number of members in the clear cluster (N_{cld}^{f}) via

$$N_{\text{clr}}^{f} \equiv \text{count}(S_{\text{clr}}), \quad \text{and,} \quad N_{\text{cld}}^{f} \equiv \text{count}(S_{\text{cld}}) \tag{6}$$

Supposing g is a placeholder that can be replaced with "clr" or "cld", $\text{count}\left(S_g\right)$ counts the number of elements in the set S_g. The parameters of Eq. (5) can then be estimated via

$$\overline{\boldsymbol{\psi}_{\boldsymbol{g}}^{\boldsymbol{f}}} \equiv \frac{1}{N_g^f} \sum_{n \in S_g} \boldsymbol{\psi}_{\boldsymbol{n}}^{\boldsymbol{f}}, \quad \overline{\boldsymbol{P}_{\boldsymbol{g}}^{\boldsymbol{f}}} \equiv \frac{1}{N_g^f - 1} \sum_{n \in S_g} \left(\boldsymbol{\psi}_{\boldsymbol{n}}^{\boldsymbol{f}} - \overline{\boldsymbol{\psi}_{\mathbf{g}}^{\boldsymbol{f}}}\right)\left(\boldsymbol{\psi}_{\boldsymbol{n}}^{\boldsymbol{f}} - \overline{\boldsymbol{\psi}_{\mathbf{g}}^{\boldsymbol{f}}}\right)^{\top}, \text{ and, } w_g^f \equiv \frac{N_g^f}{N_{\text{clr}}^f + N_{\text{cld}}^f}. \tag{7}$$

Note that the BGEnKF does not require any explicit estimate of the large matrices $\boldsymbol{P}_{\text{cld}}^{\boldsymbol{f}}$ and $\boldsymbol{P}_{\text{clr}}^{\boldsymbol{f}}$. Instead, like the typical serially assimilating EnKF, the BGEnKF only requires calculating a column of these matrices. This will be discussed in Text S4.

To assimilate y^o into $\left\{\boldsymbol{\psi}_{\mathbf{1}}^{\boldsymbol{f}}, \ldots, \boldsymbol{\psi}_{\boldsymbol{N_E}}^{\boldsymbol{f}}\right\}$, consider Bayes' rule:

$$p\left(\boldsymbol{\psi}|y^o\right) = \frac{p\left(\boldsymbol{\psi}\right)\; p\left(y^o|\boldsymbol{\psi}\right)}{p\left(y^o\right)} \tag{8}$$

where the marginal $p\left(y^o\right)$ normalizes the numerator of Eq. (8) [*e.g.*, Lorenc (1986)]. As we will show later, this normalization property is central to deriving the posterior weights of the clear and cloudy posterior kernels. Note that though the normalization property is used in the derivation, there is no need to explicit compute $p\left(y^o\right)$ at all in the BGEnKF algorithm.

If we assume Gaussian observation errors, the observation likelihood $p\left(y^o|\boldsymbol{\psi}\right)$ can be written as

$$p\left(y^o|\boldsymbol{\psi}\right) \equiv \mathcal{G}\left(\boldsymbol{H}\boldsymbol{\psi};\quad y^o, \sigma^{o2}\right) \tag{9}$$

where σ^{o2} is the observation error variance and $\boldsymbol{H}$ is a matrix that extracts the simulated observation from $\boldsymbol{\psi}$. Specifically, if y^o corresponds to the (N_x+m)-th element in $\boldsymbol{\psi}$, $\boldsymbol{H}$ is an $1 \times N_\psi$ matrix of the form

$$\boldsymbol{H} \equiv \begin{bmatrix} 0 & 0 & \ldots & 0 & 1 & 0 & \ldots & 0 & 0 \end{bmatrix}$$

where the only non-zero element (unity) is the (N_x+m)-th element.

Before proceeding further, note that the observation likelihoods for IR-BTs are not strictly Gaussian. The associated observation errors are known to be dependent on the presence/absence of clouds in the observed atmospheric columns (Geer & Bauer, 2011; Harnisch et al., 2016; Minamide & Zhang, 2017; Otkin et al., 2018). Furthermore, IR-BT values are bounded. Nonetheless, the successes seen in assimilating IR-BTs with EnKFs suggest that the imperfect Gaussian observation likelihood assumption is at least somewhat functional (Otkin, 2012; F. Zhang et al., 2016; Honda et al., 2018; Minamide & Zhang, 2018; Y. Zhang et al., 2018; Otkin & Potthast, 2019; F. Zhang et al., 2019; Geer et al., 2019; Chan, Zhang, et al., 2020; Jones et al., 2020; ?, ?; Hartman et al., 2021; Y. Zhang et al., 2021). We will thus proceed with the assumption that the observation likelihood is Gaussian.

For the ease of future reference, we will sketch out the main steps to derive the posterior pdf. Combining the bi-Gaussian forecast pdf [Eq. (5)] with the Gaussian observation likelihood [Eq. (9)] through Bayes rule [Eq. (8)] will result in

$$\begin{aligned} p\left(\boldsymbol{\psi}|y^o\right) = & w_{\text{clr}}^f\, \mathcal{G}\left(\boldsymbol{\psi};\quad \overline{\boldsymbol{\psi}_{\text{clr}}^{\boldsymbol{f}}}, \boldsymbol{P}_{\text{clr}}^{\boldsymbol{f}}\right) \mathcal{G}\left(\boldsymbol{H}\boldsymbol{\psi};\quad y^o, \sigma^{o2}\right) / p\left(y^o\right) \\ & + w_{\text{cld}}^f\, \mathcal{G}\left(\boldsymbol{\psi};\quad \overline{\boldsymbol{\psi}_{\text{cld}}^{\boldsymbol{f}}}, \boldsymbol{P}_{\text{cld}}^{\boldsymbol{f}}\right) \mathcal{G}\left(\boldsymbol{H}\boldsymbol{\psi};\quad y^o, \sigma^{o2}\right) / p\left(y^o\right) \end{aligned} \tag{10}$$

To proceed further, a well-known property is used: the multiplication of two Gaussian pdfs results in a scaled Gaussian pdf. This property is foundational to EnKFs (Evensen, 1994; Burgers et al.,

1998; Houtekamer & Mitchell, 2001; Anderson, 2001; Bishop et al., 2001; Whitaker & Hamill, 2002; Tippett et al., 2003; Hunt et al., 2007). In this situation, for the term associated with cluster g [*e.g.*, Anderson and Anderson (1999)],

$$\mathcal{G}\left(\boldsymbol{\psi};\ \overline{\boldsymbol{\psi}_{\boldsymbol{g}}^{\boldsymbol{f}}}, \boldsymbol{P}_{\boldsymbol{g}}^{\boldsymbol{f}}\right)\ \mathcal{G}\left(\boldsymbol{H\psi};\ y^o, \sigma^{o2}\right) = \alpha_g\, \mathcal{G}\left(\boldsymbol{\psi};\ \overline{\boldsymbol{\psi}_{\boldsymbol{g}}^{\boldsymbol{a}}}, \boldsymbol{P}_{\boldsymbol{g}}^{\boldsymbol{a}}\right) \tag{11}$$

where $\overline{\boldsymbol{\psi}_{\boldsymbol{g}}^{\boldsymbol{a}}}$ represents the analyzed average state of cluster g, $\boldsymbol{P}_{\boldsymbol{g}}^{\boldsymbol{a}}$ represents the analyzed covariance matrices of said cluster, and α_g is a scaling factor. $\overline{\boldsymbol{\psi}_{\boldsymbol{g}}^{\boldsymbol{a}}}$ and $\boldsymbol{P}_{\boldsymbol{g}}^{\boldsymbol{a}}$ are related to $\overline{\boldsymbol{\psi}_{\boldsymbol{g}}^{\boldsymbol{f}}}$ and $\boldsymbol{P}_{\boldsymbol{g}}^{\boldsymbol{f}}$ via the Kalman filter (KF) equations [*e.g.*, Lorenc (1986)]

$$\overline{\boldsymbol{\psi}_{\boldsymbol{g}}^{\boldsymbol{a}}} = \overline{\boldsymbol{\psi}_{\boldsymbol{g}}^{\boldsymbol{f}}} + \boldsymbol{K}_{\boldsymbol{g}}\left(y^o - \boldsymbol{H}\overline{\boldsymbol{\psi}_{\boldsymbol{g}}^{\boldsymbol{f}}}\right), \quad \text{and,} \quad \boldsymbol{P}_{\boldsymbol{g}}^{\boldsymbol{a}} = \left(\boldsymbol{I} - \boldsymbol{K}_{\boldsymbol{g}}\boldsymbol{H}\right)\boldsymbol{P}_{\boldsymbol{g}}^{\boldsymbol{f}}, \tag{12}$$

where $\boldsymbol{K}_{\boldsymbol{g}}$ is the Kalman gain matrix for cluster g. $\boldsymbol{K}_{\boldsymbol{g}}$ can be computed via

$$\boldsymbol{K}_{\boldsymbol{g}} \equiv \boldsymbol{P}_{\boldsymbol{g}}^{\boldsymbol{f}}\boldsymbol{H}^\top\left(\boldsymbol{H}\,\boldsymbol{P}_{\boldsymbol{g}}^{\boldsymbol{f}}\boldsymbol{H}^\top + \sigma^{o2}\right)^{-1} = \frac{\mathrm{Cov}\left(\boldsymbol{\psi}_{\boldsymbol{g}}^{\boldsymbol{f}},\, \boldsymbol{H\psi}_{\boldsymbol{g}}^{\boldsymbol{f}}\right)}{\mathrm{Var}\left(\boldsymbol{H\psi}_{\boldsymbol{g}}^{\boldsymbol{f}}\right) + \sigma^{o2}} \tag{13}$$

where

$$\mathrm{Cov}\left(\boldsymbol{\psi}_{\boldsymbol{g}}^{\boldsymbol{f}},\, \boldsymbol{H\psi}_{\boldsymbol{g}}^{\boldsymbol{f}}\right) \equiv \frac{1}{N_g^f - 1}\sum_{n_g \in S_g}\left(\boldsymbol{H\psi}_{\boldsymbol{n}}^{\boldsymbol{f}} - \boldsymbol{H}\overline{\boldsymbol{\psi}_{\boldsymbol{g}}^{\boldsymbol{f}}}\right)\left(\boldsymbol{\psi}_{\boldsymbol{n}}^{\boldsymbol{f}} - \overline{\boldsymbol{\psi}_{\boldsymbol{g}}^{\boldsymbol{f}}}\right),$$

$$\mathrm{Var}\left(\boldsymbol{H\psi}_{\boldsymbol{g}}^{\boldsymbol{f}}\right) \equiv \frac{1}{N_g^f - 1}\sum_{n_g \in S_g}\left(\boldsymbol{H\psi}_{\boldsymbol{n}}^{\boldsymbol{f}} - \boldsymbol{H}\overline{\boldsymbol{\psi}_{\boldsymbol{g}}^{\boldsymbol{f}}}\right)^2,$$

and n_g is a dummy index that iterates over the member indices contained in S_g. The scaling factor α_g in Eq. (11) can be shown to be [*e.g.*, Anderson and Anderson (1999)]:

$$\alpha_g = \mathcal{G}\left(y^o;\ \boldsymbol{H}\,\overline{\boldsymbol{\psi}_{\boldsymbol{g}}^{\boldsymbol{f}}},\ \sigma^{o2} + \boldsymbol{H}\boldsymbol{P}_{\boldsymbol{g}}^{\boldsymbol{f}}\boldsymbol{H}^\top\right). \tag{14}$$

Note that $\boldsymbol{H}\,\boldsymbol{\psi}_{\boldsymbol{n}}^{\boldsymbol{f}}$, $\boldsymbol{H}\,\overline{\boldsymbol{\psi}_{\boldsymbol{g}}^{\boldsymbol{f}}}$, and $\mathrm{Var}\left(\boldsymbol{H\psi}_{\boldsymbol{g}}^{\boldsymbol{f}}\right)$ are scalars. Furthermore, if y^o corresponds to the $(N_x + m)$-th element of $\boldsymbol{\psi}$, then $\mathrm{Cov}\left(\boldsymbol{\psi}_{\boldsymbol{g}}^{\boldsymbol{f}},\, \boldsymbol{H\psi}_{\boldsymbol{g}}^{\boldsymbol{f}}\right)$ is equal to the $(N_x + m)$-th column of $\boldsymbol{P}_{\boldsymbol{g}}^{\boldsymbol{f}}$.

Substituting Eq. (11) into Eq. (10) and results in

$$p\left(\boldsymbol{\psi}|y^o\right) = \frac{w_{\mathrm{clr}}^f\,\alpha_{\mathrm{clr}}\,\mathcal{G}\left(\boldsymbol{\psi};\ \overline{\boldsymbol{\psi}_{\mathrm{clr}}^{\boldsymbol{a}}}, \boldsymbol{P}_{\mathrm{clr}}^{\boldsymbol{a}}\right) + w_{\mathrm{cld}}^f\,\alpha_{\mathrm{cld}}\,\mathcal{G}\left(\boldsymbol{\psi};\ \overline{\boldsymbol{\psi}_{\mathrm{cld}}^{\boldsymbol{a}}}, \boldsymbol{P}_{\mathrm{cld}}^{\boldsymbol{a}}\right)}{p\left(y^o\right)}. \tag{15}$$

Since $p\left(y^o\right)$ normalizes Eq. (15), then,

$$\begin{aligned} p\left(y^o\right) &= \int_{R^{N_\psi}}\left\{w_{\mathrm{clr}}^f\,\alpha_{\mathrm{clr}}\,\mathcal{G}\left(\boldsymbol{\psi};\ \overline{\boldsymbol{\psi}_{\mathrm{clr}}^{\boldsymbol{a}}}, \boldsymbol{P}_{\mathrm{clr}}^{\boldsymbol{a}}\right) + w_{\mathrm{cld}}^f\,\alpha_{\mathrm{cld}}\,\mathcal{G}\left(\boldsymbol{\psi};\ \overline{\boldsymbol{\psi}_{\mathrm{cld}}^{\boldsymbol{a}}}, \boldsymbol{P}_{\mathrm{cld}}^{\boldsymbol{a}}\right)\right\}\mathrm{d}^{N_\psi}\boldsymbol{\psi} \\ &= w_{\mathrm{clr}}^f\,\alpha_{\mathrm{clr}} + w_{\mathrm{cld}}^f\,\alpha_{\mathrm{cld}} \end{aligned} \tag{16}$$

where $\int_{R^{N_\psi}}\{\cdot\}\,\mathrm{d}^{N_\psi}\boldsymbol{\psi}$ is an infinite N_ψ-dimensional volume integral of $\{\cdot\}$ over the N_ψ-dimensional space that $\boldsymbol{\psi}$ lives in [*i.e.*, an R^{N_ψ} space]. Substituting the marginal [Eq. (16)] back into Bayes' rule [Eq. (15)] gives us the following bi-Gaussian posterior pdf

$$p\left(\boldsymbol{\psi}|y^o\right) = w_{\mathrm{clr}}^a\,\mathcal{G}\left(\boldsymbol{\psi};\ \overline{\boldsymbol{\psi}_{\mathrm{clr}}^{\boldsymbol{a}}}, \boldsymbol{P}_{\mathrm{clr}}^{\boldsymbol{a}}\right) + w_{\mathrm{cld}}^a\,\mathcal{G}\left(\boldsymbol{\psi};\ \overline{\boldsymbol{\psi}_{\mathrm{cld}}^{\boldsymbol{a}}}, \boldsymbol{P}_{\mathrm{cld}}^{\boldsymbol{a}}\right) \tag{17}$$

where

$$w^a_{\text{clr}} = \frac{w^f_{\text{clr}}\,\alpha_{\text{clr}}}{w^f_{\text{clr}}\,\alpha_{\text{clr}} + w^f_{\text{cld}}\,\alpha_{\text{cld}}}, \quad \text{and}, \quad w^a_{\text{cld}} = \frac{w^f_{\text{cld}}\,\alpha_{\text{cld}}}{w^f_{\text{clr}}\,\alpha_{\text{clr}} + w^f_{\text{cld}}\,\alpha_{\text{cld}}}. \tag{18}$$

Like the EnKF, the BGEnKF will update the forecast ensemble to become consistent with the posterior bi-Gaussian pdf [Eq. (17)].

4. Text S4: Detailed description of the three-stage BGEnKF algorithm

The BGEnKF's updates to the ensemble is done through a three-stage update process (illustrated in the main text's Figure 1). In order of execution, these stages are: 1) the double EnKF stage, 2) the shrinking cluster member deletion stage, and 3) the expanding cluster member resampling stage. An outline of this three-stage BGEnKF update procedure can be found at the end of this section.

The double EnKF stage

The first stage [Figure 1(a)] is to represent the KF updates to the clusters' mean states and covariance matrices. We can thus use the ensemble square root filter of Whitaker and Hamill (2002) (EnSRF) to update each cluster's members. The EnSRF update equation (Whitaker & Hamill, 2002) for members in cluster g is

$$\boldsymbol{\psi}^{\boldsymbol{a}}_{\boldsymbol{n_g}} = \boldsymbol{\psi}^{\boldsymbol{f}}_{\boldsymbol{n_g}} + \boldsymbol{K_g}\left(y^o - \boldsymbol{H}\overline{\boldsymbol{\psi_g}}\right) - \phi_g \boldsymbol{K_g}\left(\boldsymbol{H}\boldsymbol{\psi}^{\boldsymbol{f}}_{\boldsymbol{n_g}} - \boldsymbol{H}\overline{\boldsymbol{\psi}^{\boldsymbol{f}}_{\boldsymbol{g}}}\right) \quad \forall \;\; n_g \in S_g. \tag{19}$$

The Kalman gain matrix of cluster g ($\boldsymbol{K_g}$) can be computed via Eq. (13). ϕ_g is the EnSRF's square-root modification factor (Whitaker & Hamill, 2002), which can be computed via

$$\phi_g \equiv \left\{ 1 + \sqrt{\frac{\sigma^{o2}}{\sigma^{o2} + \text{Var}\left(\boldsymbol{H}\boldsymbol{\psi}^{\boldsymbol{f}}_{\boldsymbol{g}}\right)}} \right\}^{-1}. \tag{20}$$

Note that the EnSRF-based cluster update equations can be replaced with those from the two-step ensemble adjustment Kalman filter (EAKF) of Anderson (2003). This is because the two filters have mathematically identical ensemble member update procedures.

The member deletion stage

In the second and third stages of the BGEnKF (Figure 1(b & c)), the number of ensemble members in each cluster (*i.e.*, cluster sizes) is updated to be consistent with the cluster's posterior weight [Eq. (18)]. The post-BGEnKF size of cluster g (N^a_g) can be determined by

$$N^a_g \equiv \text{round}\left(N_E * W_g\right) \tag{21}$$

where $\text{round}(\cdot)$ indicates rounding $\cdot$ to the nearest integer.

If the size of a cluster is reduced by the assimilation of y^o, we will delete members from said cluster (Figure 1(b)). The number of members to be deleted N_{del} is defined as

$$N_{\text{del}} \equiv \begin{cases} N^f_{\text{clr}} - N^a_{\text{clr}} & \text{if } N^a_{\text{clr}} < N^f_{\text{clr}}, \\ \\ N^f_{\text{cld}} - N^a_{\text{cld}} & \text{if } N^a_{\text{cld}} < N^f_{\text{cld}}. \end{cases} \tag{22}$$

For simplicity, we will delete the members with the smallest N_{del} forecast-simulated observation perturbations. Since the deletion will cause the cluster's mean state to deviate from the theoretical mean state [Eq. (12)], we will recenter the remaining members around the theoretical value. Note that no heuristic adjustments were made to mitigate the changes in the cluster's sample covariance matrix due to the deletion process. This is because it is impossible to prevent such changes in practical situations [for $N_E < N_\psi$, the rank of the pre-deletion sample covariance matrix is guaranteed to be higher than the rank of the post-deletion sample covariance matrix; Chan, Anderson, and Chen (2020)].

The resampling stage

If the size of one cluster is reduced by the assimilation of y^o, the other cluster's size will increase to compensate for the reduction. This ensures that the total number of ensemble members is unchanged. To do so, the expanding cluster's ensemble members are resampled. The expanding cluster's sample mean state and sample covariance matrix should not be altered by resampling.

The computationally efficient resampling strategy proposed in Chan, Anderson, and Chen (2020) is to resample within the extended state subspace spanned by the expanding cluster's ensemble members (henceforth referred to as the subspace resampling strategy). This is the easiest to formulate in terms of the perturbations of the expanding cluster's members. Supposing that the subscript "pre" denotes expanding cluster quantities before resampling, we can compute the pre-resampling perturbations $\left\{ \boldsymbol{\psi}_{\boldsymbol{n}}^{\boldsymbol{a}'} | n \in S_{\text{pre}} \right\}$ via

$$\boldsymbol{\psi}_{\boldsymbol{n}}^{\boldsymbol{a}'} \equiv \boldsymbol{\psi}_{\boldsymbol{n}}^{\boldsymbol{a}} - \overline{\boldsymbol{\psi}_{\textbf{pre}}^{\boldsymbol{a}}} \quad \forall \;\; n \in S_{\text{pre}} \tag{23}$$

where $\overline{\boldsymbol{\psi}_{\textbf{pre}}^{\boldsymbol{a}}}$ is the expanding cluster's mean state and S_{pre} is the set of member indices in the expanding cluster before resampling.

The central idea of the subspace resampling strategy is to construct a new set of perturbations via linear combinations of the pre-resampling perturbations. We will denote all post-resampling expanding cluster quantities with the subscript "post". Let S_{post} denote the set of member indices in the post-resampling expanding cluster. S_{post} thus includes the member indices in S_{pre} and the indices of the members deleted in the deletion stage. If we represent the set of post-resampling perturbation vectors as $\left\{ \boldsymbol{\psi}_{\boldsymbol{n^*}}^{\boldsymbol{a^*}} | n^* \in S_{\text{post}} \right\}$, the strategy's central idea can then be mathematically expressed as

$$\boldsymbol{\psi}_{\boldsymbol{n^*}}^{\boldsymbol{a^*}} \equiv \sum_{n \in S_{\text{pre}}} \boldsymbol{\psi}_{\boldsymbol{n}}^{\boldsymbol{a}'} \; T_{n,n^*} \quad \forall \quad n^* \in S_{\text{post}}$$

where T_{n,n^*} is a to-be-determined scalar factor controlling how the n-th pre-resampling perturbation contributes to the n^*-th post-resampling perturbation. This linear combination idea can be more succinctly expressed as

$$\boldsymbol{\Psi}_{\textbf{post}} \equiv \boldsymbol{\Psi}_{\textbf{pre}} \boldsymbol{T}. \tag{24}$$

Here, $\boldsymbol{\Psi}_{\textbf{pre}}$ is a matrix where each column contains a pre-resampling perturbation, and $\boldsymbol{\Psi}_{\textbf{post}}$ is a matrix where each column contains a post-resampling perturbation. Supposing the pre-resampling cluster size is denoted by N_{pre} and the post-resampling cluster size is denoted by N_{post}, then $\boldsymbol{\Psi}_{\textbf{pre}}$ is an $N_\psi \times N_{\text{pre}}$ matrix and $\boldsymbol{\Psi}_{\textbf{post}}$ is an $N_\psi \times N_{\text{post}}$ matrix. If we denote the ℓ-th member index in S_{pre} as $n_{\text{pre},\ell}$, and likewise for the ℓ-th member index in S_{post}, we can explicitly write out $\boldsymbol{\Psi}_{\textbf{pre}}$ and $\boldsymbol{\Psi}_{\textbf{post}}$:

$$\begin{aligned} \boldsymbol{\Psi}_{\textbf{pre}} &\equiv \left[\boldsymbol{\psi}_{\boldsymbol{n}_{\textbf{pre},\mathbf{1}}}^{\boldsymbol{a}'} \;\; \boldsymbol{\psi}_{\boldsymbol{n}_{\textbf{pre},\mathbf{2}}}^{\boldsymbol{a}'} \;\; \cdots \;\; \boldsymbol{\psi}_{\boldsymbol{n}_{\textbf{pre},\boldsymbol{N}_{\textbf{pre}}}}^{\boldsymbol{a}'} \right], \\ \boldsymbol{\Psi}_{\textbf{post}} &\equiv \left[\boldsymbol{\psi}_{\boldsymbol{n}_{\textbf{post},\mathbf{1}}}^{\boldsymbol{a^*}} \;\; \boldsymbol{\psi}_{\boldsymbol{n}_{\textbf{post},\mathbf{2}}}^{\boldsymbol{a^*}} \;\; \cdots \;\; \boldsymbol{\psi}_{\boldsymbol{n}_{\textbf{post},\boldsymbol{N}_{\textbf{post}}}}^{\boldsymbol{a^*}} \right]. \end{aligned} \tag{25}$$

Finally, $\boldsymbol{T}$ is an $N_{\text{pre}} \times N_{\text{post}}$ matrix containing all of the T_{n,n^*} values [*i.e.*, element (n, n^*) of $\boldsymbol{T}$ is equal to T_{n,n^*}].

$\boldsymbol{T}$ should be constructed such that the post-resampling perturbations have a mean of zero and have a covariance matrix equal to that of pre-resampling perturbations. As discussed in Chan, Anderson, and Chen (2020), there are an infinite number of possible $\boldsymbol{T}$'s that satisfy these two conditions. Following the discussions and heuristic arguments in Chan, Anderson, and Chen (2020), we chose to use

$$\boldsymbol{T} \equiv \begin{bmatrix} k\boldsymbol{I}_{\boldsymbol{N}_{\text{pre}}-\boldsymbol{N}^*_{\text{new}}} & \boldsymbol{0}_{(\boldsymbol{N}_{\text{pre}}-\boldsymbol{N}^*_{\text{new}})\times \boldsymbol{N}^*_{\text{new}}} & \boldsymbol{0}_{(\boldsymbol{N}_{\text{pre}}-\boldsymbol{N}^*_{\text{new}})\times \boldsymbol{N}_{\text{new}}} \\ \\ \boldsymbol{0}_{\boldsymbol{N}^*_{\text{new}}\times(\boldsymbol{N}_{\text{pre}}-\boldsymbol{N}^*_{\text{new}})} & \boldsymbol{I}_{\boldsymbol{N}^*_{\text{new}}} & \boldsymbol{E} \end{bmatrix} \tag{26}$$

where

$$N_{\text{new}} \equiv N_{\text{post}} - N_{\text{pre}}, \quad \text{and,} \quad N^*_{\text{new}} \equiv \begin{cases} N_{\text{new}} - 1 \;\forall\; N_{\text{new}} \leq N_{\text{pre}} \\ \\ N_{\text{pre}} \qquad \text{otherwise} \end{cases} . \tag{27}$$

Furthermore, for arbitrary integers η and μ, $\boldsymbol{I}_{\boldsymbol{\eta}}$ is an $\eta \times \eta$ identity matrix, $\boldsymbol{0}_{\boldsymbol{\eta}\times\boldsymbol{\mu}}$ is an $\eta \times \mu$ matrix of zeros. k is the following scalar inflation factor

$$k \equiv \sqrt{\frac{N_{\text{new}} + N_{\text{pre}} - 1}{N_{\text{pre}} - 1}} \qquad \text{(note that } k \geq 1\text{)}. \tag{28}$$

The matrix $\boldsymbol{E}$ in Eq. (26) is an $N^*_{\text{new}} \times N_{\text{new}}$ matrix that will be defined shortly. Since $N^*_{\text{new}} < N_{\text{new}}$ [see Eq. (27)], $\boldsymbol{E}$ is a rectangular matrix with more columns than rows. Note that whenever $N_{\text{new}} > N_{\text{pre}}$, the $k\boldsymbol{I}_{\boldsymbol{N}_{\text{pre}}-\boldsymbol{N}^*_{\text{new}}}$ component vanishes from $\boldsymbol{T}$. Furthermore, whenever $N_{\text{new}} = 1$, the $\boldsymbol{I}_{\boldsymbol{N}^*_{\text{new}}}$ and $\boldsymbol{E}$ components vanish from $\boldsymbol{T}$.

Our choice of $\boldsymbol{E}$ is nearly identical to that of Chan, Anderson, and Chen (2020):

$$\boldsymbol{E} \equiv \frac{k-1}{N_{\text{new}}} \boldsymbol{1}_{\boldsymbol{N}^*_{\text{new}}\times \boldsymbol{N}_{\text{new}}} + \boldsymbol{L}_{\boldsymbol{E}} (\boldsymbol{L}_{\boldsymbol{W}})^{-1} \boldsymbol{W}. \tag{29}$$

Here, $\boldsymbol{1}_{\boldsymbol{N}^*_{\text{new}}\times \boldsymbol{N}_{\text{new}}}$ denotes an $N^*_{\text{new}} \times N_{\text{new}}$ matrix whose elements are all set to unity. Furthermore, $\boldsymbol{W}$ is an $N^*_{\text{new}} \times N_{\text{new}}$ matrix of the form

$$\boldsymbol{W} \equiv \begin{bmatrix} \boldsymbol{I}_{\boldsymbol{N}^*_{\text{new}}} & \boldsymbol{0}_{\boldsymbol{N}^*_{\text{new}}\times(\boldsymbol{N}_{\text{new}}-\boldsymbol{N}^*_{\text{new}})} \end{bmatrix} - \frac{1}{N_{\text{new}}} \boldsymbol{1}_{\boldsymbol{N}^*_{\text{new}}\times \boldsymbol{N}_{\text{new}}}. \tag{30}$$

Supposing that Chol $(\boldsymbol{S})$ denotes the Cholesky decomposition of an arbitrary symmetric matrix $\boldsymbol{S}$, following appendix B of Chan, Anderson, and Chen (2020), we define

$$\boldsymbol{L}_{\boldsymbol{W}} \equiv \text{Chol}\left(\boldsymbol{W}\boldsymbol{W}^\top\right), \tag{31}$$

and

$$\boldsymbol{L}_{\boldsymbol{E}} \equiv \text{Chol}\left(\frac{N_{\text{new}}}{N_{\text{pre}} - 1} \boldsymbol{I}_{\boldsymbol{N}^*_{\text{new}}} - \frac{(k-1)^2}{N_{\text{new}}} \boldsymbol{1}_{\boldsymbol{N}^*_{\text{new}}\times \boldsymbol{N}^*_{\text{new}}} \right). \tag{32}$$

The only difference between the current formulation of $\boldsymbol{E}$ and that of Chan, Anderson, and Chen (2020) lies in the $\boldsymbol{W}$ matrix. In Chan, Anderson, and Chen (2020), $\boldsymbol{W}$ is created from vectors of random white noise. For the ease of parallelization and to ensure replicability (*i.e.*, reruns of

the BGEnKF should give the same result), we replaced that stochastic $\boldsymbol{W}$ generation procedure with a deterministic one [*i.e.*, Eq. (30)].

As discussed in Chan, Anderson, and Chen (2020), the resampled perturbations generated by the $\boldsymbol{T}$ defined in Eq. (26) has the property of preserving the pre-resampling perturbations (up to an inflation factor). More specifically, the first $N_{\text{pre}} - N^*_{\text{new}}$ resampled perturbations are inflated versions of the first $N_{\text{pre}} - N^*_{\text{new}}$ pre-resampling perturbations. The next N^*_{new} resampled perturbations are copies of N^*_{new} of the pre-resampling perturbations. Finally, the remaining N_{new} resampled perturbations are linear combinations of the copied perturbations.

Outline of three-stage BGEnKF update procedure to assimilate an observation

The outline of the three-stage BGEnKF procedure is as follows. Note that this outline assumes that the members have already been sorted into the clear and cloudy clusters (see the last paragraph of Text S2 for how members are sorted into the two clusters).

Stage 1: Double EnKF [illustrated in Figure 1(a)]

1. Do $g =$ clr, cld
   (i) For cluster g, compute the Kalman gain [$\boldsymbol{K_g}$; Eq. (13)] and square-root modification factor [ϕ_g; Eq. (20)].
   (ii) Evaluate Eq. (19) for every ensemble member in cluster g.

Stage 2: Shrinking cluster member deletion [illustrated in Figure 1(b)]

1. Evaluate Eq. (21) to determine the targeted cluster sizes after assimilating the observation
2. If $N^a_{\text{clr}} < N^f_{\text{clr}}$, the clear cluster will be considered as the shrinking cluster.
3. If $N^a_{\text{cld}} < N^f_{\text{cld}}$, the cloudy cluster will be considered as the shrinking cluster.
4. If no shrinking cluster has been identified, terminate the current stage.
5. Compute N_{del} using Eq. (22).
6. Compute the current mean state of the shrinking cluster.
7. Delete the members with the smallest N_{del} forecast-simulated observation perturbations within the shrinking cluster.
8. Compute the mean state of the remaining members in the shrinking cluster.
9. Subtract the mean computed in step 8 from the mean computed in step 6.
10. Add the difference computed in step 9 to each of the remaining members in the shrinking cluster to recenter said members on the pre-deletion shrinking cluster mean state.

Stage 3: Resample expanding cluster members [illustrated in Figure 1(c)]

1. Evaluate Eq. (21) to determine the targeted cluster sizes after assimilating the observation
2. If $N^a_{\text{clr}} > N^f_{\text{clr}}$, the clear cluster will be considered as the expanding cluster.
3. If $N^a_{\text{cld}} > N^f_{\text{cld}}$, the cloudy cluster will be considered as the expanding cluster.
4. If no expanding cluster has been identified, terminate the current stage.
5. Compute N_{new} and N^*_{new} using Eq. (27).
6. Compute the expanding cluster's mean state vector.
7. Construct the expanding cluster's perturbation vectors via Eq. (23).
8. Construct matrix $\boldsymbol{W}$ by evaluating Eq. (30).
9. Construct $\boldsymbol{L_W}$ and $\boldsymbol{L_E}$ by evaluating Eqs. (31) and (32).
10. Construct $\boldsymbol{E}$ by evaluating Eq. (29).
11. Construct $\boldsymbol{T}$ by evaluating Eq. (26).
12. Evaluate Eq. (24) to resample the expanding cluster perturbations.
13. Add the expanding cluster's mean state (computed in step 6) to the resampled perturbations to construct the resampled expanding cluster ensemble members.

5. Text S5: Outline of the BGEnKF algorithm serial filtering workflow

We will now outline the workflow of the serially assimilating BGEnKF algorithm (illustrated in Figure S2). The serially assimilating BGEnKF algorithm executes the following list of steps.

1. Construct an ensemble of forecast $\boldsymbol{\psi}$ vectors from the prior ensemble using Eq. (4).
2. Select the first observation by setting $m = 1$.
3. Employ the adaptive observation error inflation (AOEI) of Minamide and Zhang (2017) to mitigate representation errors.
4. Extract an ensemble of ξ values from the ensemble of $\boldsymbol{\psi}$ vectors that corresponds to the m-th observation site. Members whose extracted ξ values are smaller than 1 g/m^2 are considered as clear members. The remaining members are considered as cloudy members.
5. Run through the heuristic checks in the main text's sections 2.5.2 and 2.5.3 to determine whether the BGEnKF or its single-kernel form (essentially an EnKF) should be used.
6. If any of the heuristic checks in step 5 fail, put all ensemble members into the clear cluster.
7. Apply the three-stage algorithm described in Text S4 to update the ensemble of $\boldsymbol{\psi}$ vectors.
8. Localize the $\boldsymbol{\psi}$ vector updates using the main text's Eq. (7).
9. Increment m (*i.e.*, $m \leftarrow m + 1$).
10. If there are unassimilated observations remaining, go back to step 3.
11. Extract the model states contained in the ensemble of $\boldsymbol{\psi}$ vectors, output said model states, and terminate the algorithm.

To implement this algorithm with parallelization on the PSU-EnKF system, we employed the low-latency computing cluster strategy proposed by Anderson and Collins (2007). Specifically, every process will receive a sub-domain's worth of model state variables, an entire domain of observation and simulated observation values, and an entire domain of ξ values. To assimilate an observation, each process will then update its sub-domain of model state variables, all of its simulated observations, and all of its ξ values. As such, no inter-process communications are needed within the serial assimilation loop.

6. Text S6: On generalizing the BGEnKF algorithm to handle more clusters

The BGEnKF algorithm can be generalized to handle an arbitrary number of ensemble clusters (*e.g.*, a three-cluster GMM-EnKF). We did not use more than two clusters in this study because this study is a first approach to testing a cluster GMM-EnKF with a realistic weather model. Furthermore, using more clusters means that each cluster will contain fewer members. With smaller cluster sizes, the deleterious impacts of sampling errors on each cluster's sample statistics are likely stronger. Considering the small ensemble size that will be used in this first-approach study (50 members), we opted to use two clusters for now.

To generalize the BGEnKF to handle N_c clusters, only a few modifications are needed: 1) the ensemble clustering method needs to be adjusted to sort the ensemble into the N_c clusters, and 2) a slightly different method would be needed to infer the posterior cluster sizes [Eq. (22)]. The latter modification is necessary because using Eq. (22) with more than 2 clusters can cause the total number of ensemble members to change. This change arises from the use of the rounding function. For instance, suppose we have 3 clusters with equal posterior weights (0.333333 each) and the ensemble size is 10. Using Eq. (22) will result in 3 members in each cluster, or 9 members in total. A different approach to convert the non-integer weights into integer cluster sizes is thus necessary for $N_c > 2$.

References

Anderson, J. L. (2001, 12). An ensemble adjustment Kalman filter for data assimilation. *Monthly Weather Review*, *129*(12), 2884–2903. Retrieved from `http://journals.ametsoc.org/doi/10.1175/1520-0493(2001)129%3C2884:AEAKFF%3E2.0.CO;2` doi: 10.1175/1520-0493(2001)129⟨2884:AEAKFF⟩2.0.CO;2

Anderson, J. L. (2003, 4). A Local Least Squares Framework for Ensemble Filtering. *Monthly Weather Review*, *131*(4), 634–642. Retrieved from `http://journals.ametsoc.org/doi/10.1175/1520-0493(2003)131<0634:ALLSFF>2.0.CO;2` doi: 10.1175/1520-0493(2003)131⟨0634:ALLSFF⟩2.0.CO;2

Anderson, J. L., & Anderson, S. L. (1999). A Monte Carlo implementation of the nonlinear filtering problem to produce ensemble assimilations and forecasts. *Monthly Weather Review*. doi: 10.1175/1520-0493(1999)127⟨2741:AMCIOT⟩2.0.CO;2

Anderson, J. L., & Collins, N. (2007, 8). Scalable Implementations of Ensemble Filter Algorithms for Data Assimilation. *Journal of Atmospheric and Oceanic Technology*, *24*(8), 1452–1463. Retrieved from `https://journals.ametsoc.org/view/journals/atot/24/8/jtech2049_1.xml` doi: 10.1175/JTECH2049.1

Anderson, J. L., Hoar, T., Raeder, K., Liu, H., Collins, N., Torn, R., & Avellano, A. (2009, 9). The data assimilation research testbed a community facility. *Bulletin of the American Meteorological Society*, *90*(9), 1283–1296. Retrieved from `https://journals.ametsoc.org/doi/10.1175/2009BAMS2618.1` doi: 10.1175/2009BAMS2618.1

Bishop, C. H., Etherton, B. J., & Majumdar, S. J. (2001, 3). Adaptive Sampling with the Ensemble Transform Kalman Filter. Part I: Theoretical Aspects. *Monthly Weather Review*, *129*(3), 420–436. Retrieved from http://journals.ametsoc.org/doi/10.1175/1520-0493(2001)129%3C0420:ASWTET%3E2.0.CO;2https://journals.ametsoc.org/view/journals/mwre/129/3/1520-0493_2001_129_0420_aswtet_2.0.co_2.xml doi: 10.1175/1520-0493(2001)129⟨0420:ASWTET⟩2.0.CO;2

Burgers, G., Jan van Leeuwen, P., Evensen, G., Van Leeuwen, P. J., & Evensen, G. (1998, 6). Analysis scheme in the ensemble Kalman filter. *Monthly Weather Review*, *126*(6), 1719–1724. Retrieved from http://journals.ametsoc.org/doi/10.1175/1520-0493(1998)126%3C1719:ASITEK%3E2.0.CO;2 doi: 10.1175/1520-0493(1998)126⟨1719:ASITEK⟩2.0.CO;2

Chan, M.-Y., Anderson, J. L., & Chen, X. (2020). An efficient bi-Gaussian ensemble Kalman filter for satellite infrared radiance data assimilation. *Monthly Weather Review*. doi: 10.1175/mwr-d-20-0142.1

Chan, M.-Y., Zhang, F., Chen, X., & Leung, L. R. (2020). Potential Impacts of Assimilating All-sky Satellite Infrared Radiances on Convection-Permitting Analysis and Prediction of Tropical Convection. *Monthly Weather Review*. doi: 10.1175/mwr-d-19-0343.1

Evensen, G. (1994). Sequential data assimilation with a nonlinear quasi-geostrophic model using Monte Carlo methods to forecast error statistics. *Journal of Geophysical Research*, *99*(C5), 10143–10162. Retrieved from https://doi.org/10.1029/94JC00572http://doi.wiley.com/10.1029/94JC00572 doi: 10.1029/94JC00572

Geer, A. J., & Bauer, P. (2011, 10). Observation errors in all-sky data assimilation. *Quarterly Journal of the Royal Meteorological Society*, *137*(661), 2024–2037. Retrieved from http://doi.wiley.com/10.1002/qj.830 doi: 10.1002/qj.830

Geer, A. J., Migliorini, S., & Matricardi, M. (2019). All-sky assimilation of infrared radiances sensitive to mid- and upper-tropospheric moisture and cloud. *Atmospheric Measurement Techniques Discussions*. doi: 10.5194/amt-2019-9

Harnisch, F., Weissmann, M., & Periáñez. (2016). Error model for the assimilation of cloud-affected infrared satellite observations in an ensemble data assimilation system. *Quarterly Journal of the Royal Meteorological Society*. doi: 10.1002/qj.2776

Hartman, C. M., Chen, X., Clothiaux, E. E., & Chan, M.-Y. (2021, 7). Improving the Analysis and Forecast of Hurricane Dorian (2019) with Simultaneous Assimilation of GOES-16 All-Sky Infrared Brightness Temperatures and Tail Doppler Radar Radial Velocities. *Monthly Weather Review*, *149*(7), 2193–2212. Retrieved from https://journals.ametsoc.org/view/journals/mwre/149/7/MWR-D-20-0338.1.xml doi: 10.1175/MWR-D-20-0338.1

Honda, T., Miyoshi, T., Lien, G. Y., Nishizawa, S., Yoshida, R., Adachi, S. A., ... Bessho, K. (2018, 1). Assimilating all-sky Himawari-8 satellite infrared radiances: A case of Typhoon Soudelor (2015). *Monthly Weather Review*, *146*(1), 213–229. doi: 10.1175/MWR-D-16-0357.1

Houtekamer, P. L., & Mitchell, H. L. (2001). A sequential ensemble Kalman filter for atmospheric data assimilation. *Monthly Weather Review*, *129*(1), 123–137. doi: 10.1175/1520-0493(2001)129⟨0123:ASEKFF⟩2.0.CO;2

Houtekamer, P. L., & Zhang, F. (2016). Review of the ensemble Kalman filter for atmospheric data assimilation. *Monthly Weather Review*, *144*(12), 4489–4532. doi: 10.1175/MWR-D-15-0440.1

Hunt, B. R., Kostelich, E. J., & Szunyogh, I. (2007, 6). Efficient data assimilation for spatiotemporal chaos: A local ensemble transform Kalman filter. *Physica D: Nonlinear Phenomena*, *230*(1-2), 112–126. Retrieved from https://linkinghub.elsevier.com/retrieve/pii/S0167278906004647 doi: 10.1016/j.physd.2006.11.008

Ide, K., Courtier, P., Ghil, M., & Lorenc, A. C. (1997). Unified notation for data assimilation: Operational, sequential and variational. *Journal of the Meteorological Society of Japan*. doi: 10.2151/jmsj1965.75.1B{\

_}181

Jones, T. A., Skinner, P., Yussouf, N., Knopfmeier, K., Reinhart, A., Wang, X., ... Palikonda, R. (2020, 5). Assimilation of GOES-16 Radiances and Retrievals into the Warn-on-Forecast System. *Monthly Weather Review*, *148*(5), 1829–1859. Retrieved from `https://journals.ametsoc.org/view/journals/mwre/148/5/mwr-d-19-0379.1.xml` doi: 10.1175/MWR-D-19-0379.1

Lorenc, A. (1986). Analysis methods for numerical weather prediction. *Quart J. R. Mrt. Soc.*, *112*, 1177–1194. doi: 10.1256/smsqj.47413

Meng, Z., & Zhang, F. (2007). Tests of an ensemble Kalman filter for mesoscale and regional-scale data assimilation. Part II: Imperfect model experiments. *Monthly Weather Review*, *135*(4), 1403–1423. doi: 10.1175/MWR3352.1

Minamide, M., & Zhang, F. (2017). Adaptive observation error inflation for assimilating all-Sky satellite radiance. *Monthly Weather Review*, *145*(3), 1063–1081. doi: 10.1175/MWR-D-16-0257.1

Minamide, M., & Zhang, F. (2018). Assimilation of all-sky infrared radiances from Himawari-8 and impacts of moisture and hydrometer initialization on convection-permitting tropical cyclone prediction. *Monthly Weather Review*, *146*(10), 3241–3258. doi: 10.1175/MWR-D-17-0367.1

Otkin, J. A. (2012). Assessing the impact of the covariance localization radius when assimilating infrared brightness temperature observations using an ensemble kalman filter. *Monthly Weather Review*. doi: 10.1175/MWR-D-11-00084.1

Otkin, J. A., & Potthast, R. (2019). Assimilation of All-Sky seviri infrared brightness temperatures in a regional-scale ensemble data assimilation system. *Monthly Weather Review*. doi: 10.1175/MWR-D-19-0133.1

Otkin, J. A., Potthast, R., & Lawless, A. S. (2018). Nonlinear bias correction for satellite data assimilation using taylor series polynomials. *Monthly Weather Review*. doi: 10.1175/MWR-D-17-0171.1

Tippett, M. K., Anderson, J. L., Bishop, C. H., Hamill, T. M., & Whitaker, J. S. (2003, 7). Ensemble Square Root Filters. *Monthly Weather Review*, *131*(7), 1485–1490. Retrieved from `http://journals.ametsoc.org/doi/10.1175/1520-0493(2003)131%3C1485:ESRF%3E2.0.CO;2` doi: 10.1175/1520-0493(2003)131⟨1485:ESRF⟩2.0.CO;2

Whitaker, J. S., & Hamill, T. M. (2002, 7). Ensemble data assimilation without perturbed observations. *Monthly Weather Review*, *130*(7), 1913–1924. Retrieved from `http://journals.ametsoc.org/doi/10.1175/1520-0493(2002)130%3C1913:EDAWPO%3E2.0.CO;2` doi: 10.1175/1520-0493(2002)130⟨1913:EDAWPO⟩2.0.CO;2

Zhang, F., Minamide, M., & Clothiaux, E. E. (2016). Potential impacts of assimilating all-sky infrared satellite radiances from GOES-R on convection-permitting analysis and prediction of tropical cyclones. *Geophysical Research Letters*, *43*(6), 2954–2963. doi: 10.1002/2016GL068468

Zhang, F., Minamide, M., Nystrom, R. G., Chen, X., Lin, S.-J., & Harris, L. M. (2019, 7). Improving Harvey Forecasts with Next-Generation Weather Satellites: Advanced Hurricane Analysis and Prediction with Assimilation of GOES-R All-Sky Radiances. *Bulletin of the American Meteorological Society*, *100*(7), 1217–1222. Retrieved from `https://journals.ametsoc.org/view/journals/bams/100/7/bams-d-18-0149.1.xml` doi: 10.1175/BAMS-D-18-0149.1

Zhang, Y., Sieron, S. B., Lu, Y., Chen, X., Nystrom, R. G., Minamide, M., ... Zhang, F. (2021, 12). *Ensemble-Based Assimilation of Satellite All-Sky Microwave Radiances Improves Intensity and Rainfall Predictions for Hurricane Harvey (2017)* (Vol. 48) (No. 24). John Wiley and Sons Inc. doi: 10.1029/2021GL096410

Zhang, Y., Zhang, F., & Stensrud, D. J. (2018). Assimilating all-sky infrared radiances from GOES-16 ABI using an ensemble Kalman filter for convection-allowing severe thunderstorms prediction. *Monthly Weather Review*. doi: 10.1175/MWR-D-18-0062.1

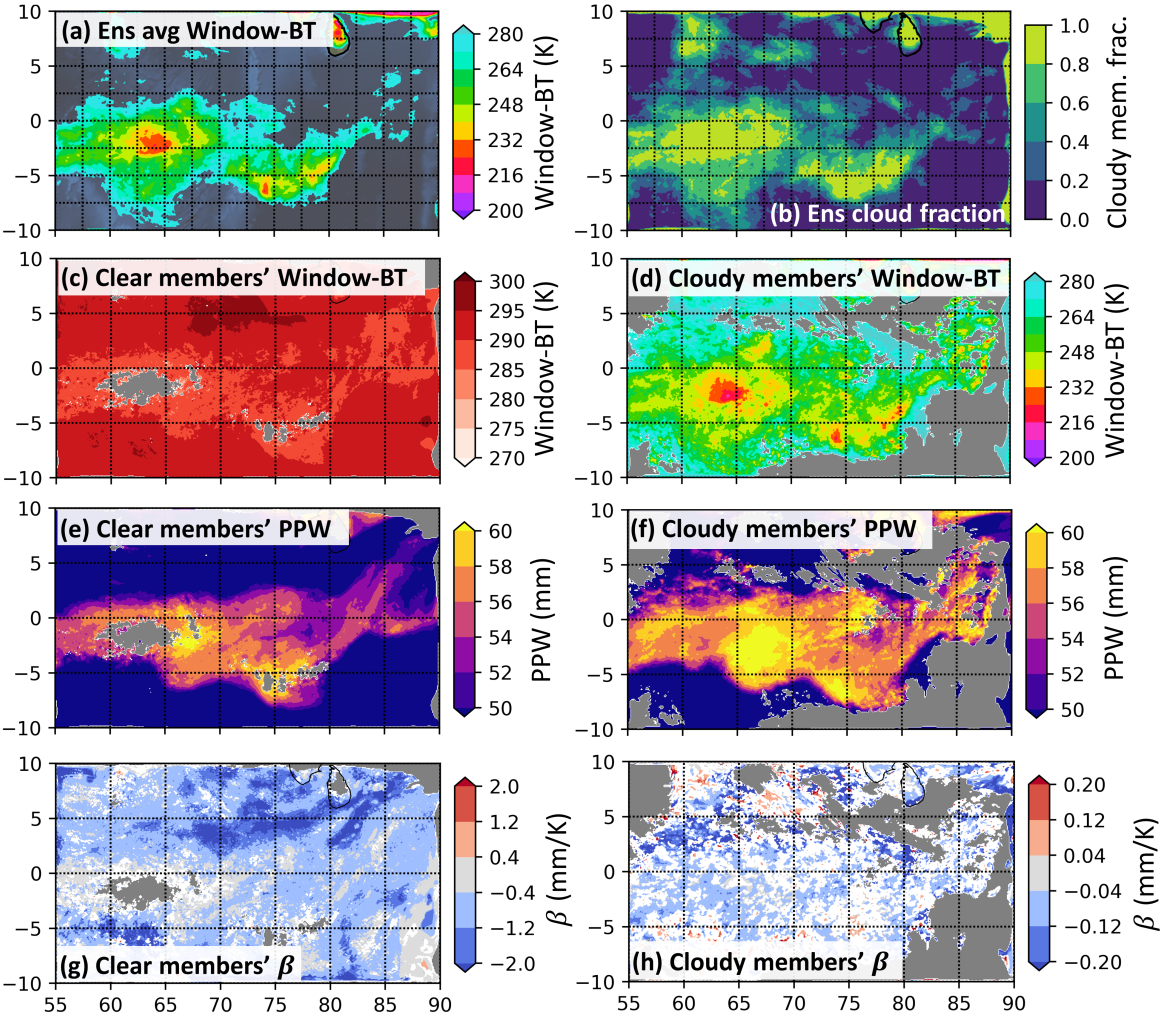

Figure S1: Latitude-longitude plots of various ensemble statistics at 1200 UTC on 15 October 2011 to illustrate the differences between clear and cloudy sky members at every model column. These quantities are generated using the 50-member ensemble described in the main text. The y-axes indicate latitude (degrees North), and the x-axes indicate longitude (degrees East). The plotted quantities are: the prior ensemble mean Window-BT (a), the fraction of cloudy member columns in the prior ensemble at every grid column (b), the mean Window-BTs of clear member columns (c), the mean Window-BT of cloudy member columns (d), the mean pseudo precipitable water (PPW) for clear member columns (e), the mean PPW for cloudy member columns (f), the linear regression coefficient between Window-BT and PPW (β) for clear member columns (g), and the β values for cloudy member columns (h). The gray shadings in panels c, e & g indicate locations where there are either less than 5 clear member columns, the clear member columns' Window-BT sample variance is zero, or the clear member columns' PPW sample variance is zero. The gray shadings in panels d, f & h indicate locations where there are either less than 5 cloudy member columns, the cloudy member columns' Window-BT sample variance is zero, or the cloudy member columns' PPW sample variance is zero. The white shadings in panels g indicate areas where the clear member columns' sample correlation between PPW and Window-BT is statistically insignificant, and likewise for the white shadings in panel h.

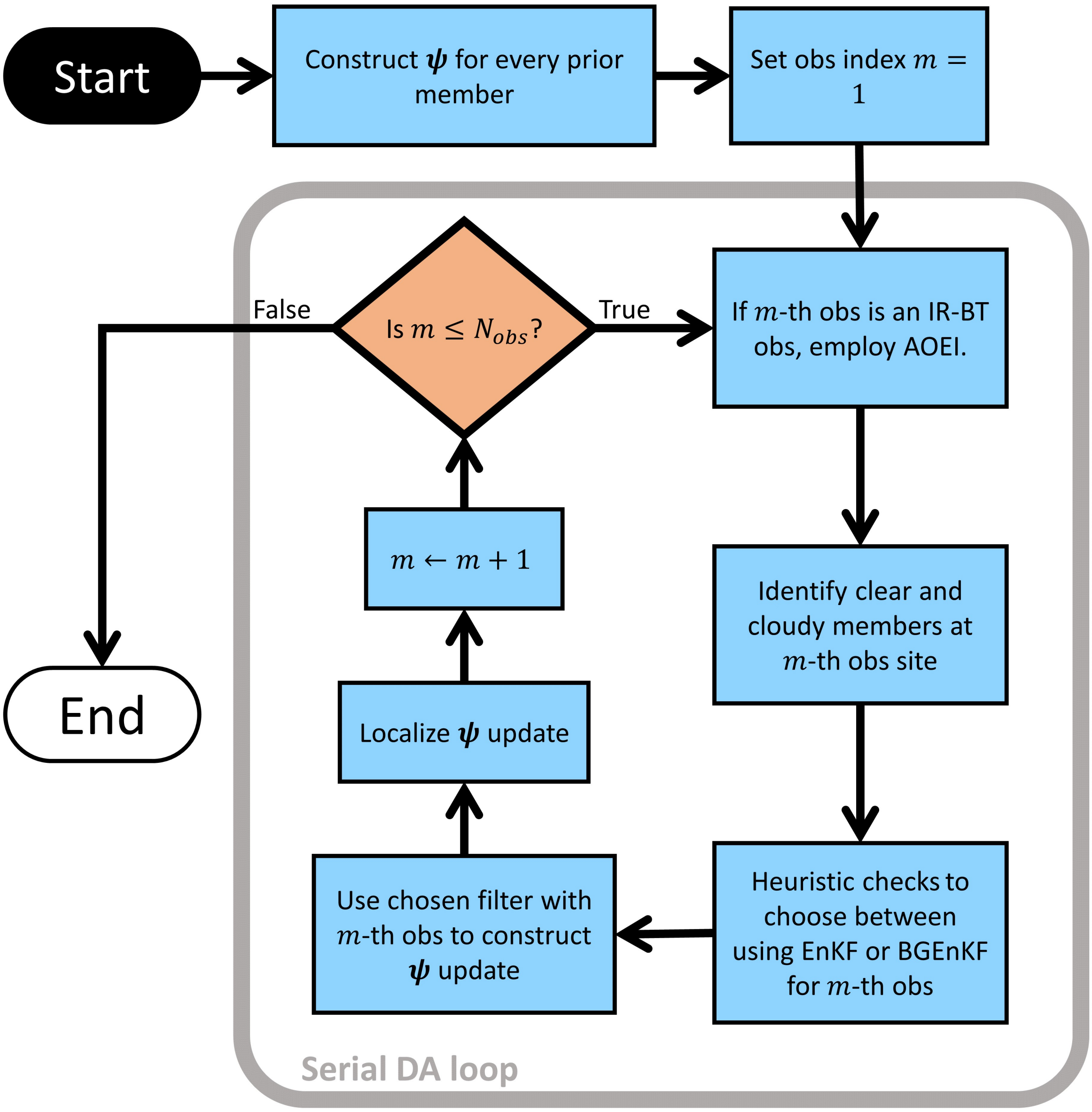

Figure S2: Workflow of the BGEnKF module implemented in the PSU DA system. "Obs" stands for "observations" and N_{obs} stands for the total number of observations. See the text for the definitions of the extended state vector ψ [Eq. (3)], the list of heuristic checks used to select between the EnKF and BGEnKF (main text section 2.5), and for a description of the BGEnKF update procedure (Text S4). The three-stage BGEnKF update procedure is illustrated in Figure 1 of the main text.
